# Travertine crystal growth ripples record the hydraulic history of ancient Rome’s Anio Novus aqueduct

**DOI:** 10.1038/s41598-022-05158-2

**Published:** 2022-01-24

**Authors:** Duncan Keenan-Jones, Davide Motta, Marcelo H. Garcia, Mayandi Sivaguru, Mauricio Perillo, Ryan K. Shosted, Bruce W. Fouke

**Affiliations:** 1 School of Historical and Philosophical Inquiry, The University of Queensland, St Lucia, QLD 4072 Australia; 2 Department of Mechanical and Construction Engineering, Northumbria University, Wynne Jones Building, Newcastle Upon Tyne, NE1 8ST UK; 3 Ven Te Chow Hydrosystems Laboratory, Department of Civil and Environmental Engineering, University of Illinois at Urbana-Champaign, 205 North Mathews Avenue, Urbana, IL 61801 USA; 4 Cytometry and Microscopy to Omics Facility, Roy J. Carver Biotechnology Center, University of Illinois Urbana-Champaign, 231 Edward L. Madigan Laboratory, 1201 W. Gregory Drive, Urbana, IL 61801 USA; 5 ExxonMobil Upstream Business Development, 22777 Springwoods Village Parkway, Spring, TX 77389 USA; 6 Department of Linguistics, University of Illinois at Urbana-Champaign, 707 S Mathews Ave., Urbana, Champaign, IL USA; 7 Department of Geology, University of Illinois at Urbana-Champaign, 1301. W. Green St., Urbana, IL 61801 USA; 8 Department of Evolution, Ecology, and Behavior, University of Illinois at Urbana-Champaign, 505 S. Goodwin Ave., Urbana, IL USA; 9 Roy J. Carver Biotechnology Center, University of Illinois at Urbana-Champaign, 1206 W. Gregory Drive, Urbana, IL 61801 USA; 10 Carl R. Woese Institute for Genomic Biology and Carl Zeiss Labs@Location Partner, University of Illinois at Urbana-Champaign, 1206 W. Gregory Drive, Urbana, IL 61801 USA

**Keywords:** Sedimentology, Civil engineering

## Abstract

Travertine crystal growth ripples are used to reconstruct the early hydraulic history of the Anio Novus aqueduct of ancient Rome. These crystalline morphologies deposited within the aqueduct channel record the hydraulic history of gravity-driven turbulent flow at the time of Roman operation. The wavelength, amplitude, and steepness of these travertine crystal growth ripples indicate that large-scale sustained aqueduct flows scaled directly with the thickness of the aqueous viscous sublayer. Resulting critical shear Reynolds numbers are comparable with those reconstructed from heat/mass transfer crystalline ripples formed in other natural and engineered environments. This includes sediment transport in rivers, lakes, and oceans, chemical precipitation and dissolution in caves, and melting and freezing in ice. Where flow depth and perimeter could be reconstructed from the distribution and stratigraphy of the travertine within the Anio Novus aqueduct, flow velocity and rate have been quantified by deriving roughness-flow relationships that are independent of water temperature. More generally, under conditions of near-constant water temperature and kinematic viscosity within the Anio Novus aqueduct channel, the travertine crystal growth ripple wavelengths increased with decreasing flow velocity, indicating that systematic changes took place in flow rate during travertine deposition. This study establishes that travertine crystal growth ripples such as those preserved in the Anio Novus provide a sensitive record of past hydraulic conditions, which can be similarly reconstructed from travertine deposited in other ancient water conveyance and storage systems around the world.

## Introduction

A reliable supply of fresh drinking water, coupled with an effective water delivery management system, are two of the essential elements required to build and maintain human civilization. Fulfilment of these basic requirements has carried power and prestige in virtually every culture throughout human history^1^. Where water has sufficiently high supersaturated concentrations of dissolved minerals (hard water), it precipitates deposits composed of calcium carbonate (CaCO_3_) limestone called *travertine*^2,3^ within water transport and storage systems. In the process, these travertine deposits preserve a record of the complexly intertwined physical, chemical, and biological processes that have influenced their deposition^2–5^. Travertine deposited during the operation of historical aqueducts has recorded changes in human activity and climate in Europe and the Middle East^6–15^, Pre-Columbian North America^16^, Central Asia^17^ and Australia^18^. While dominated by CaCO_3_ crystals that precipitate directly from the flowing aqueduct water, aqueduct travertine also contains varying but relatively minor amounts of downstream-transported sediments and plant debris^19^. This combination of travertine crystallization and sedimentation processes posed a significant ongoing problem for the maintenance of ancient aqueducts^20^. Travertine had to be regularly removed manually^19,21,22^ to prevent it from becoming too heavy for the aqueduct structural supports, while also increasing wall roughness, narrowing channel cross-sectional area, and reducing the flow capacity^23^. As a result of these relationships between aqueduct flow and travertine formation, aqueduct travertine has recently been used to reconstruct flow rates based on their spatial and temporal distribution within the archaeologically significant and protected ruins of the Anio Novus aqueduct at Roma Vecchia, Italy^23,24^.

In many natural submarine and subaerial environmental systems, downstream transport of sedimentary particles creates sediment transport morphologies called ripples and dunes that have been extensively studied^25–32^. In addition, other types of deposits called “heat/mass transfer” crystalline deposits have also been described that form ripples as a result of either heat transfer during melting and freezing in ice (e.g. ice ripples)^33–38^, or mass transfer during mineral precipitation and dissolution in caves (e.g. solution ripples^36–42^; Supplementary Information S1). Another common example of heat/mass transfer crystalline deposits are travertine microterracettes formed in terrestrial spring, river, lake, and cave hydraulic systems. In these environments, travertine microterracettes (repeated pond and dam stair-steps) form as a result of complex interactions between crystal precipitation from supersaturated aqueous solutions, changing gravity-dependent low-flow hydraulic regimes, the presence and metabolic activity of microorganisms, and a small amount of downstream sediment transport^2,43–48^.

The present study evaluates an unexpected new class of well-preserved heat/mass transfer crystalline deposits that formed during travertine precipitation on the floors, walls, and roofs of the main channel of the Anio Novus aqueduct of ancient Rome^19,23,24^ These deposits were initially reported as “ripples” in the Anio Novus travertine^19^ and later called “ripple-like” morphologies in geographically widespread aqueducts in France^13^, Istanbul and Jordan^9^, Germany^15^, and Turkey^14^. To be consistent with previous literature, the term “ripple” in the present study will be used as a descriptive morphological term, to which modifiers are added to indicate specific compositions and genetic processes of formation and deposition. Examples include “sediment transport ripples”^28,32^, “solution ripples”^42^, and even “ice ripples”^37^. Depositional morphologies observed in the present study of Anio Novus aqueduct travertine deposits will therefore be consistently described as “travertine crystal growth ripples”. This terminology confirms that the travertine ripple morphologies form from a process of constructional crystal growth directly from the flowing aqueduct water. Furthermore, this terminology reflects the distinctly different typology of travertine crystallized from complex physical, chemical, and biological mechanisms^2–5^, which is a fundamentally different process from the fluid mechanics controlling sedimentary transport ripple formation^28^. At the same time, the nomenclature “travertine crystal growth ripples” recognizes that gravity-driven, open-channel turbulent flow is also influential in convective diffusion during travertine CaCO_3_ crystal precipitation^33^. As a result, the present study combines analyses of travertine crystal growth ripple morphology, 3D floor and wall distributions within the aqueduct channel, and hydraulic modeling to reconstruct the operating conditions of the ancient Anio Novus aqueduct.

## Materials and methods

### Archeological setting

The largest and most important addition to the water supply system of ancient Rome was the unprecedented simultaneous building of the Aqua Claudia and the Anio Novus aqueducts between 38 and 52 CE^49^. The 11 aqueducts built in Rome between 312 BCE and 226 CE constituted a larger and more complex urban water supply system than any before it. Collectively, these aqueducts, 500 km in total length^50^, enabled the population density to reach unprecedented levels that rival those of modern-day urbanization^51^. Considerable municipal funds were expended in their maintenance and in some cases the aqueducts continued to be used for over a thousand years^52^.

**Figure 1:**
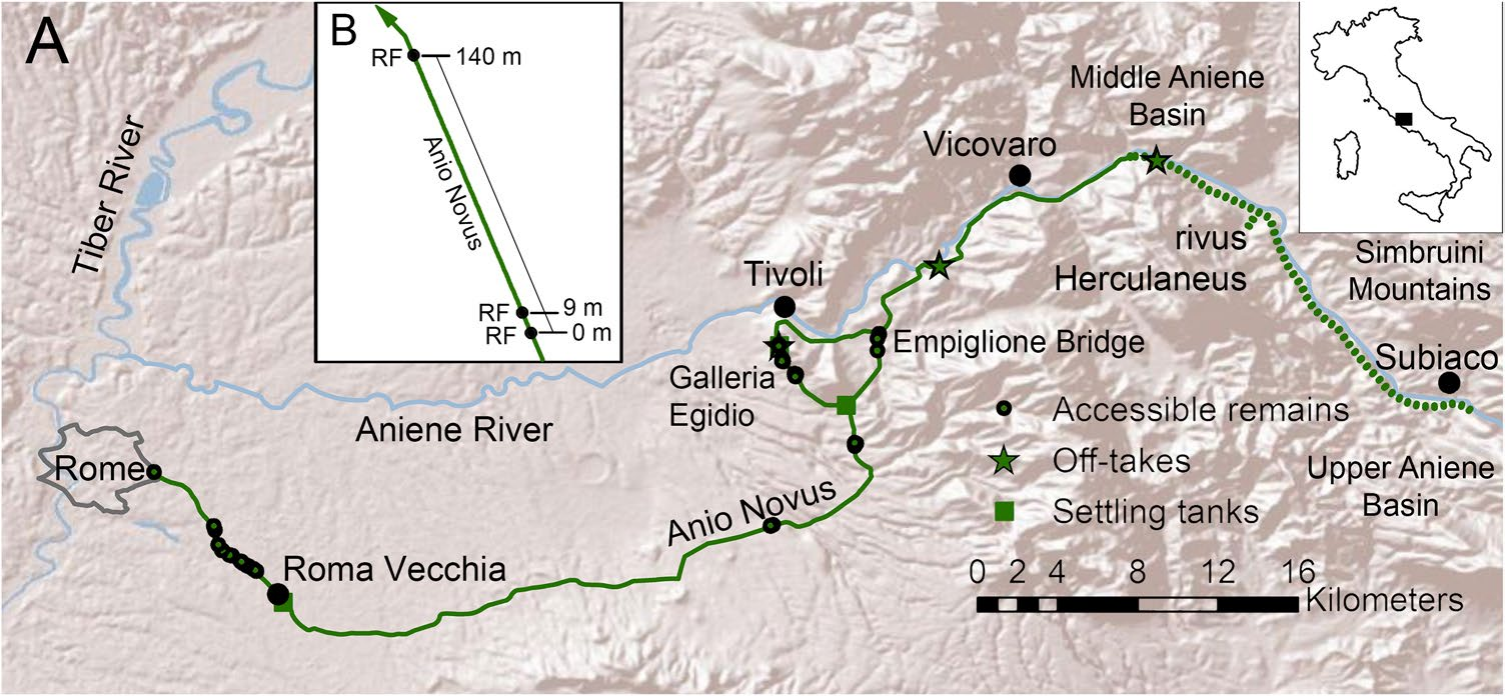
(**A**) Route of the Anio Novus aqueduct from Subiaco in the Apennines into Rome. Geographic location of the Galleria Egidio, Empiglione Bridge and Roma Vecchia sites are indicated. (**B**) Enlargement of the Anio Novus aqueduct channel at Roma Vecchia showing relative locations of the upstream (RF 0 m), intermediate (RF 9 m), and downstream (RF 140 m) sample collection locations. Modified from Keenan-Jones et al.^24^.

The Anio Novus^23,24^ was the farthest-reaching aqueduct ever built by Imperial Rome, carrying water from the Aniene (Latin: *Anio*) River and one of its tributaries, the *rivus Herculaneus,* some 87 km into the Eternal City^21^ (Fig. 1A). The Anio Novus aqueduct was fed from the Middle and Upper Basins of the Aniene River^21^, which flows through and dissolves Mesozoic (Upper Triassic to Upper Cretaceous) CaCO_3_ marine limestones comprising the Simbruini Mountains^53^. Around 98 CE, the water supply system of Rome was thoroughly described in the *De Aquis* compendium by Sextus Julius Frontinus, Rome’s water commissioner (*curator aquarum*). Because of the high carbonate supersaturation of the water, travertine reached more than 1 m in thickness at some sites along the aqueduct system^49^. The Anio Novus was the highest-elevation aqueduct along most of the Aniene valley and the second highest in elevation within the city of Rome itself^54^. Aqueduct elevation was important (Supplementary Information S2) since Roman water supply was predominantly gravity-driven and only areas below an aqueduct could be supplied with any significant volume of water^50^. Maintenance of the Anio Novus apparently ceased sometime between the fifth and eighth centuries CE^52,55^.

**Figure 2:**
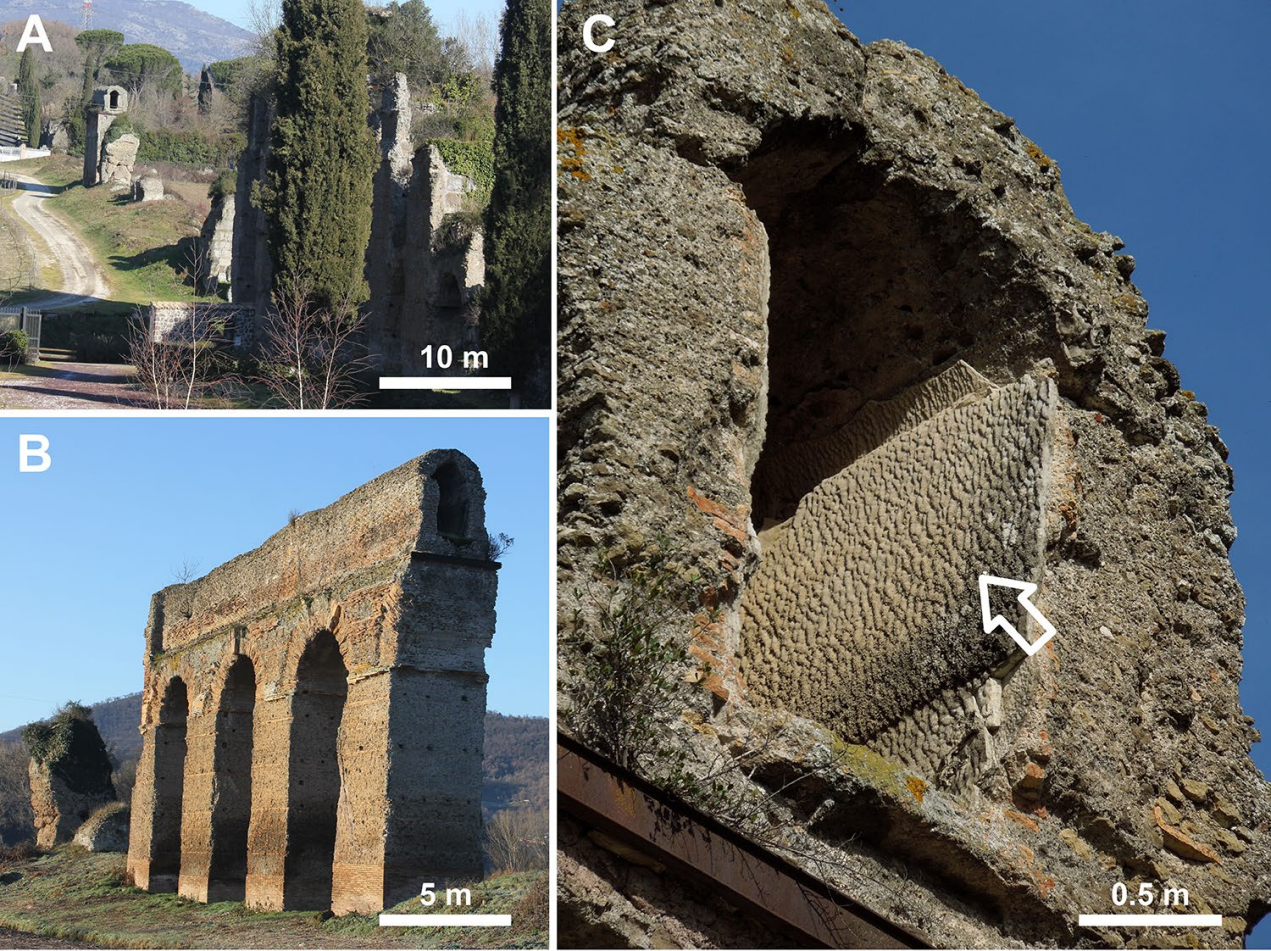
Field photographs of the Anio Novus aqueduct at Empiglione Bridge on the Tivoli Bypass (location shown in Fig. 1A). (**A**) Empilgione Bridge looking upstream. (**B**) Closeup of arcade at upstream end (top left) of (**A**), now looking downstream. (**C**). Travertine crystal growth ripples (white arrow) on the downstream vertical side wall of the arcade shown in (**B**) (looking upstream). This opening is visible in the top left of (**A**).

Sample collection for the present study was conducted under research permits granted by the Soprintendenza Speciale per il Colosseo, il Museo Nazionale Romano e l'Area Archeologica di Roma and the Soprintendenza Archeologia, Belle Arti e Paesaggio per l'Area Metropolitana di Roma, la Provincia di Viterbo e l'Etruria Meridionale. Aqueduct travertine samples were collected from three sites: (1) the underground Galleria Egidio section of the Tivoli Loop^23^ (Fig. 1A); (2) the Empiglione Bridge section on the Tivoli Bypass (Figs. 1A and 2); and (3) three locations within the site of Roma Vecchia^24^ (Fig. 1B). Travertine deposited at each of these three locations lined the channel floor, walls and sometimes roof of the Anio Novus aqueduct, exhibiting depositional characteristics that are generally consistent with previous descriptions from other Roman aqueduct systems^6,7,9,15^.

The distribution and depositional characteristics of the travertine crystal growth ripples at the Galleria Egidio and Roma Vecchia sites were used to justify the assumption of uniform flow within the Anio Novus aqueduct at these locations, which have been previously presented in detail^23,24^ (Supplementary Table S2, Supplementary Information S5.1). Similarly, the Empiglione Bridge site, which begins at the Anio Novus' channel bifurcation around Tivoli (Fig. 1A), contains a 625 m-long bridge^55^ that also provides strong evidence for uniform flow. This is because it crossed the valley of the Empiglione River by means of a straight arcade that maintained essentially constant gradients and cross-sectional geometries^49,54^. The Empiglione Bridge sample sites in the present study were located ~ 200 m downstream of the bifurcation and 400 m upstream of the end of the bridge (Fig. 2). The only known aqueduct junction on the bridge is an offtake to a cistern located 230 m downstream of the measured cross section, which is not expected to have affected the uniform flow profile significantly. The stone piers and arches comprising the bridge date to the original construction of the Anio Novus^49,55^. The three arches of the bridge portion from which the Empiglione Bridge samples were collected, have been attributed to repairs conducted under the Roman emperor Hadrian^49,55^.

At Roma Vecchia, the Anio Novus aqueduct emerges from the ground and was built directly upon the Aqua Claudia aqueduct, which was itself supported by an arcade up to 10 m in height (Fig. 2A, B)^21,24^. Three age-equivalent upstream-to-downstream samples of travertine at Roma Vecchia were collected from the floor of a 140 m-long continuous section of the Anio Novus aqueduct^12^. These included an upstream 0 m location (sample number RNRV3-2A), an intermediate 9 m location (sample number RNRV3-3A) and a downstream 140 m location (sample number RNRV1-2A; Fig. 1B).

### Aqueduct travertine field sample collection

Travertine samples collected from all sites at Galleria Egidio, Empiglione Bridge and Roma Vecchia (Figs. 1, 2) were systematically photographed, measured, and described before careful removal using a hammer and small, clean, well-sharpened chisels composed of hardened steel. Each travertine sample was labelled (e.g., sample number, upstream–downstream context and flow direction determined by contextual orientation the sample within the aqueduct channel), bagged, and shipped in a padded container to the Microscopy and Imaging Core Facility of the Carl R. Woese Institute for Genomic Biology (IGB) at the University of Illinois Urbana-Champaign (Illinois). Samples were cut on a clean water-cooled diamond embedded tile saw in an orientation parallel to the upstream–downstream flow direction of the channel. Samples were then thoroughly washed with deionized water, polished, dried in a clean room, and photographed with a Nikon SLR D7000 digital camera (Nikon, Japan).

### Petrographic thin-section preparation and optical microscopy

Billets cut from the three travertine samples collected at Roma Vecchia (Fig. 1B) were prepared by Wagner Petrographic (Linden, Utah) as Petropoxy impregnated, doubly polished, uncovered, 25 µm-thick sections mounted on standard-sized petrographic glass slides. Optical microscopy of these thin sections was completed on a Hamamatsu Nanozoomer digital slide scanner using a 20 × 0.75 NA UPlansApo objective at a pixel resolution of 0.23 µm under a brightfield (transmitted light) modality. Further detailed descriptions of the light, laser, electron, and x-ray microscopy techniques used to analyze the Anio Novus aqueduct travertine samples are presented in Sivaguru et al. (2022).

### Hydraulic measurements

**Figure 3:**
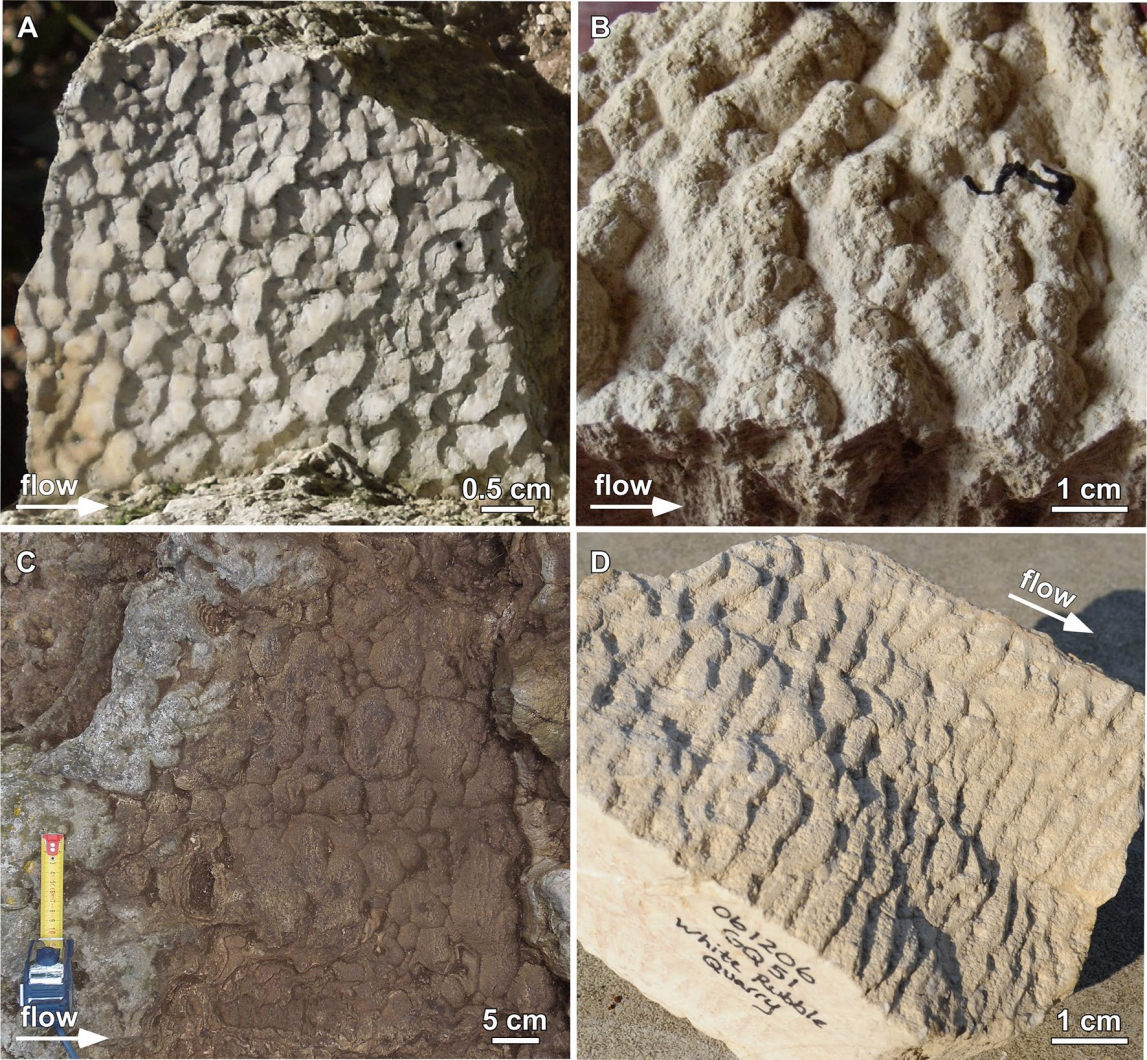
Comparison of different types of travertine crystal growth ripple morphologies deposited in the Anio Novus aqueduct with travertine microterracette morphologies deposited in hot-spring drainage systems. (**A**–**C**) Anio Novus travertine crystal growth ripple morphologies formed at the three Roma Vecchia sample sites (Fig. 1B). (**A**) Linguoid travertine crystal growth ripples at RF 0 m; (**B**) sinuous ripples at RF 9 m. (**C**) Hummocky travertine crystal growth ripples at RF 140 m. (**D**) Pleistocene-age Distal-Slope Facies travertine microterracette morphologies deposited in the quarries of Gardiner, Montana (modified from^2^).

**Figure 4:**
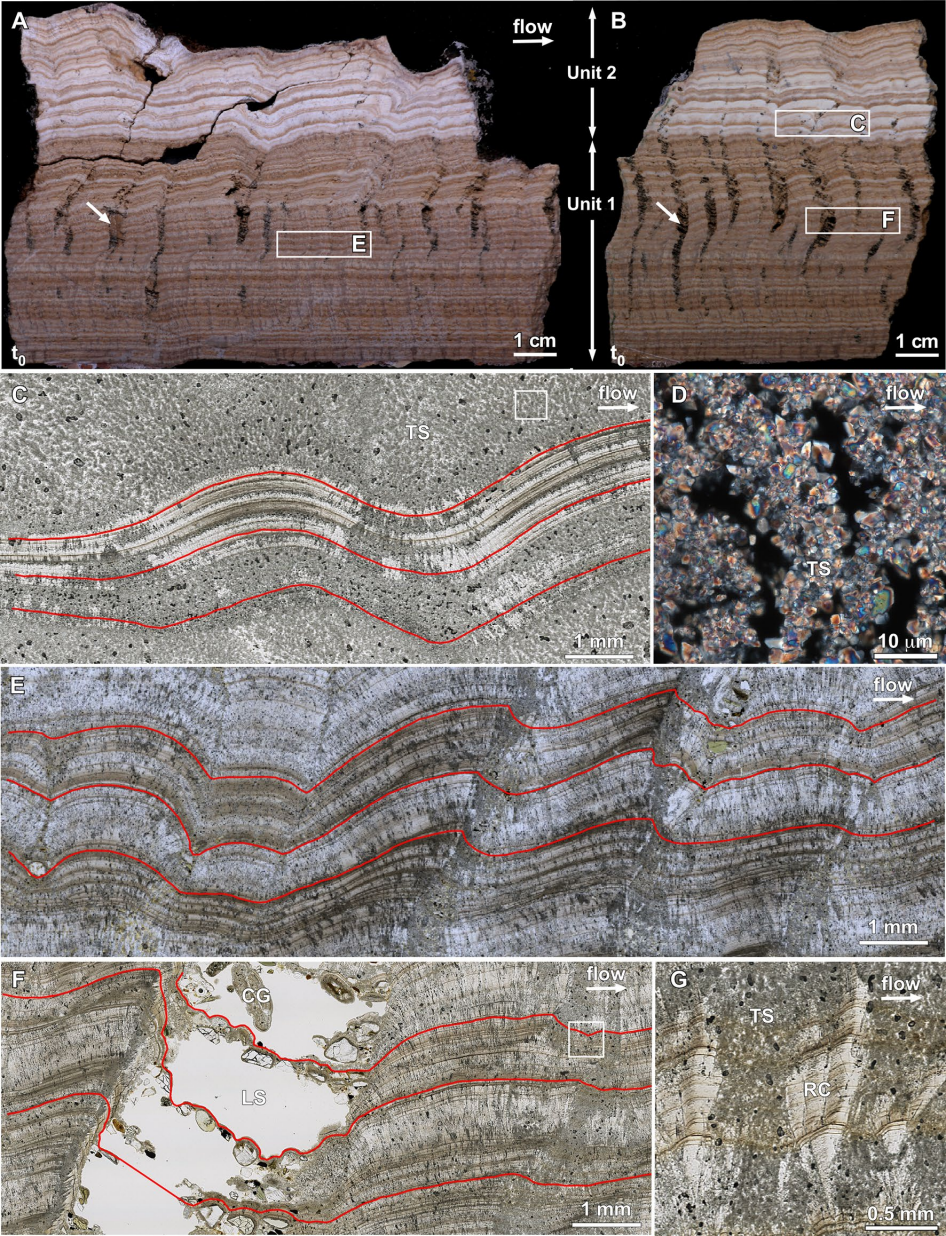
Dark–light laminae stratigraphy of travertine hand-sample cross-sections deposited within the Anio Novus aqueduct. Samples were collected from the upstream RF 0 m (Sample Number: RNRV3-2A; **A**) and downstream RF 140 m (Sample Number: RNRV1-2A; **B**) sites at Roma Vecchia (Fig. 1B). Travertine depositional Units 1 and 2 and downstream flow direction (white arrows) are indicated. Modified from^12^. (**A**) and (**B**). Standard reflected light hand-sample photographs of the face of vertical cross-sections oriented parallel to the downstream flow direction. Depositional age was determined via correlation of the t_0_ contact surface between the underlying mortar and overlying travertine. Lee sands were deposited on the lee side of each travertine crystal growth ripple morphology (white arrows in **A** and **B**). (**C**) Nanozoomer brightfield (transmitted plane light) thin-section photomicrograph (white box C in B) showing travertine shrubs (TS). Tracings of three representative linguoid travertine crystal growth ripple morphology cross-sections are shown (red lines). (**D**) Polarized light high-resolution widefield photomicrograph (enlargement of white box in **C**) of dendritically branching aggregates (shrubs) of 1 to 3 µm-diameter euhedral calcite crystals. (**E**) Nanozoomer brightfield thin-section photomicrograph (Box E in A). Tracings of three representative linguoid travertine crystal growth ripple morphology cross-sections are shown (red lines). (**F**) Nanozoomer brightfield thin-section photomicrograph (white box in **F**). Tracings of three representative linguoid travertine crystal growth ripple morphology cross-sections are shown (red lines). Labels indicate regions of lee sands (LS) with coated grains (CG) that were partially removed during cutting and thin-section preparation. (**G**) Enlargement of white box in (**F**). Diagenetic replacement radiaxial calcite (RC) crystals form upward radiating bundles that crosscut the original travertine shrubs (TS) and alternating dark–light laminae stratigraphy.

Each of the Anio Novus travertine crystal growth ripple morphology samples analyzed in the present study were carefully photographed, measured and marked to record their precise position, three-dimensional (3D) distribution, and upstream to downstream orientation within the aqueduct channel. This included measurement of the wetted perimeter and cross-sectional flow area of the aqueduct channel at each site^23^, techniques presented in^24^. Reconstruction of the flow rate of the ancient Anio Novus aqueduct water was done by assuming uniform flow, which is justified and accurate if: (1) the channel cross-section, gradient and direction are roughly constant; and (2) the sample site is away from hydraulic control points, such as sluice gates, junctions, or branches. Many of ancient Rome’s aqueduct channels cannot be studied because they have been destroyed, or are inaccessible due their height above ground, depth underground, or because the channel is filled with sediment and soil. Although these factors make the widespread regional mapping and analysis of aqueduct travertine ripples challenging, three sites were identified within the Anio Novus aqueduct (Fig. 1) that meet these criteria and exhibit mm- to cm-scale travertine crystal growth ripples (Figs. 3 and 4).

The slope of the Anio Novus aqueduct channel floor at Roma Vecchia was calculated using previously reported topographic elevation^54^, while distances between each elevation point were calculated using ArcGIS^23,24^. In addition, previously determined channel and travertine geometry data were used for both Galleria Egidio and Roma Vecchia^23,24^. The channel at Empiglione Bridge could not be accessed due to its height above ground level. A Leica total station (Leica Camera AG, Wetzlar, Germany) was used to measure the geometry of the channel and the travertine crystal growth ripple morphologies (Supplementary Fig. S5) and then processed in the SolidWorks 2015 Premium × 64 SP5.0 program (Dassault Systèmes SE, Vélizy-Villacoublay, France).

### Characterization of travertine crystal growth ripples

Variations in the morphological characteristics of the Anio Novus travertine crystal growth ripples through time (i.e., along vertical stratigraphic cross-sections at each geographic location) and space (i.e., at different sample locations along the channel flow path) were qualitatively and quantitatively investigated. Bottom-to-top analyses of the travertine crystal growth ripple layers through the entirety of the 8 cm-thick stratigraphy, which records changes through time at three locations at Roma Vecchia, was evaluated using digital image analysis of individual stratigraphic horizons (described below). These were further evaluated with direct hand-sample and thin-section images analyses and measurement using open-source NIH ImageJ software. A total of 21 specific travertine crystal growth ripple layers (stratigraphic horizons), each representing the travertine-water growth interface at any given specific point in time, were evaluated. These were consistently identified within digital scans of Roma Vecchia hand-sample cross-sections by tracking individual dark laminae. The shape of each layer of travertine crystal growth ripples was then determined using the Livewire function in the MIPAV program (National Institutes of Health, USA, https://mipav.cit.nih.gov/). Finally, each horizon was analysed in MATLAB (MathWorks, Natick, MA, USA) with the results collated and compared with direct sample measurement performed in MIPAV, Adobe Photoshop and Microsoft Excel (Microsoft, Redmond, WA, USA).

Three primary geometric characteristics of the travertine crystal growth ripples were analysed including wavelength ($\lambda$), amplitude (*a* = 0.5 × ripple height [Δ]), and steepness (= Δ/$\lambda$). Ripple wavelengths were analysed by Fourier transform. The Lomb-Scargle algorithm was implemented with Welch-Overlapped Segment Averaging (WOSA, 50% overlapping periodograms) to produce Fourier transforms of each stratigraphic horizon. Peak identifications from the resulting power spectrum were tested for significance against either: (1) a white-noise (random) null hypothesis; or (2) an autoregressive red-noise null hypothesis where each point depends on the point before it. The red noise character is defined as decreasing spectral power with increasing wavenumber. Confidence limits (= (1 − (1/n), where n is the number of points sampled by Mipav along the ripples)^56^ were calculated using 1000 Monte Carlo simulations.

Four different proxies (described in Supplementary Information S5.3) for amplitude were then calculated from the ripple horizons for RF 140 m (Fig. 1B). Ground truthing (measurement of amplitude of ripples in NIH software ImageJ) showed that the most accurate proxy was the mean of the displacement of the background-subtracted horizon (Supplementary Fig. S6) and this proxy was also calculated for the other samples.

The aim of investigating variations in travertine crystal growth ripples at the three sites along the Anio Novus aqueduct channel flow path (Fig. 1B) was to evaluate the relation between the flow properties and the ripple wavelength in the flow direction. A single stratigraphic horizon composed of travertine crystal growth ripples was investigated at each of the three sites. These were: (1) the “latest flow” at Empiglione Bridge (the rippled surface visible in Fig. 2C); (2) the “early flow” at Galleria Egidio; and (3) the “latest flow” at Roma Vecchia. The “latest flow” at Roma Vecchia is 19 cm above (and hence deposited later than) the top of the Unit 2 (Fig. 4), and located 1 m upstream (Fig. 3D). At each stratigraphic position, representative depositional surfaces and a two-dimensional (2D) vertical cross-section of the travertine samples, each from different locations on the wetted perimeter, was analysed to estimate a boundary-averaged wavelength. More than 30 wavelength measurements for each site were made and the Sauter mean wavelength (${\overline{\lambda }}_{32}$) estimated, following accepted practice^58^.

Travertine samples chosen for analysis were deposited directly onto the uppermost surface of the mortar lining of the Anio Novus aqueduct channel and exhibited complete and continuous stratigraphic sequences (i.e., no evidence of significant disruption, erosion, or dissolution during deposition). At Empiglione Bridge, measurements of ripple wavelengths of the travertine surface were made at the vault (1.9 m above the floor) and two locations (at 1.3 m and 0.9 m above the floor) on the left-hand wall (when facing downstream, Fig. 2C), using the Leica total station as described above. At Galleria Egidio (Supplementary Fig. S7) and Roma Vecchia (Supplementary Fig. S8), ripple wavelengths in the centre of the channel floor were measured from photographs taken perpendicular to the travertine surface. Wavelengths and ripple height were measured on scans of polished cross-sections of travertine crystal growth ripple stratigraphy from the “latest flow” at Empiglione Bridge (where samples were collected immediately downstream of the measured section, Supplementary Fig. S9), from the wall of the “early flow” at Galleria Egidio (Supplementary Fig. S10) and the floor of the “latest flow” at Roma Vecchia (Supplementary Fig. S11). These samples were all less than 3 wavelengths in length in the downstream direction, therefore ripples were measured directly in ImageJ rather than by Fourier transform.

## Results

### Travertine stratigraphy and crystal growth ripple morphology

Detailed analyses of the crystalline structure, sedimentology, stratigraphy, and diagenetic alteration of the three travertine samples within the Anio Novus aqueduct channel at Roma Vecchia (Fig. 1B) are presented in Sivaguru et al.^12^. The following summary provides a fundamentally important depositional and diagenetic context for characterization of the Anio Novus travertine crystal growth ripple morphology in the present study. The Anio Novus aqueduct travertine deposited at each of the three Roma Vecchia sites (Fig. 1B) is composed of: (1) the top of the underlying Roman mortar on the floor of the aqueduct; (2) the time-zero (t_0_) surface comprising the contact between the underlying mortar and the overlying travertine; and (3) an 8 cm-thick deposit of aqueduct travertine composed of an underlying 5 cm-thick Unit 1 and an overlying 3 cm-thick Unit 2^12^. Travertine crystal growth ripples observed in three-dimensions (3D) on in situ bedding surfaces and hand samples (Fig. 3), as well as throughout all vertical slices of collected samples (Fig. 4), are described in the following. Age-equivalency of the three Roma Vecchia travertine samples (Fig. 1B) was established via correlation of the t_0_ surface, their compositional and stratigraphic consistency in crystalline texture, color, thickness and layering, and the lack of evidence for any later alteration, disturbance, or dissolution during possible Roman maintenance (Fig. 4)^12^.

The Unit 1 and 2 travertine deposits (Fig. 4A, B) are composed of two types of calcium carbonate (CaCO_3_) morphologies, which include: (1) original 50 to 100 µm-tall dendritically branching aggregates of small (1–3 µm-diameter) euhedral calcite crystals (Fig. 4C; called *shrubs*); and (2) variably sized (100’s µm to 10’s mm) diagenetic replacement *radiaxial calcite* crystals that form upward radiating bundles that crosscut the alternating dark–light laminae stratigraphy but do not influence the original travertine crystal growth ripple morphologies (Fig. 4D, E)^12^. Both types contain stratigraphic sequences of interlayered dark brown and light beige laminae (< 10 to 100 µm-thick), formed by organic matter entrapped during original crystallization and deposition (Fig. 4)^12^. Unit 1 travertine is composed of high-frequency interlayering of 0.1 to 1 mm-thick dark brown laminae and light beige laminae that are generally planar, yet sometimes exhibit low angle angular unconformities (Fig. 4A, B). While the thickness of the dark brown laminae remains relatively consistent in Unit 1 and 2 (100’s µm-thick to ~ 1 mm-thick), the light beige laminae are significantly thicker in Unit 2 (~ 1–3 mm-thick; Fig. 4A, B).

**Figure 5:**
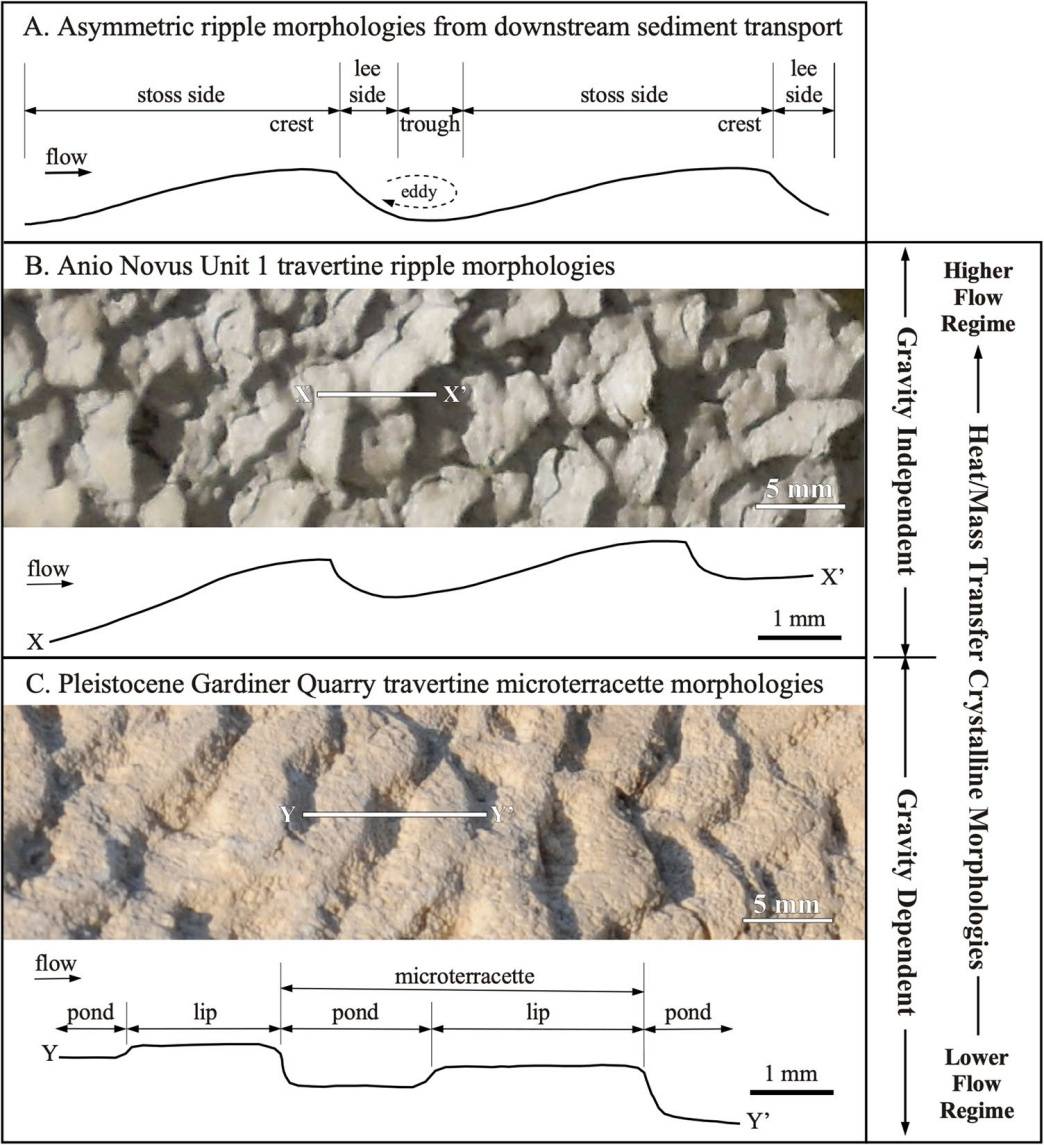
Comparison between Anio Novus travertine crystal growth ripple morphologies and Gardiner Quarry travertine microterracette morphologies. (**A**) Terminology used to describe the geomorphology of asymmetric ripples formed by downstream hydraulic transport of sedimentary grains^28,30,31^. (**B**) Enlargement of Anio Novus Unit 1 travertine linguoid travertine crystal growth ripple morphologies shown in Fig. 3A. X–X’ is the line of section shown for the morphology cross-section tracing shown in Fig. 4E. (**C**) Enlargement of Pleistocene Gardiner Quarry Distal-Slope Facies travertine microterracette morphology shown in Fig. 3D. Y–Y’ is the line of section shown in the morphology cross-section tracing.

Unit 1 exhibits travertine crystal growth ripples with stoss, crest, lee and trough geomorphologies (Figs. 4 and 5; Supplementary Videos S1 and S2)^12,28,30,31^. On bedding surfaces observed in the field, in hand sample and in thin section (Figs. 3, 4, and 5B, C), these travertine crystal growth ripples exhibit wavelengths generally increasing from mm scale near the bottom to cm scale near the top and are morphologically consistent with linguoid, sinuous and hummocky ripples observed in sedimentary transport ripples^12,28,30,31^. As these layers of travertine crystal growth ripples accumulated vertically, the position of the crests and troughs intermittently preferentially accumulated in a downstream direction (*prograded*) and at other times in an upstream direction (*retrograded*), forming zig-zag stratigraphic patterns in 2D vertical sections (Figs. 3 and 4)^12^ (Supplementary Videos S1 and S2, described in more detail in Supplementary Information S3.1). In Unit 2, the travertine crystal growth ripple wavelengths are generally on the cm scale and larger than those in Unit 1,forming sinuous and hummocky sedimentary transport ripples (Figs. 4 and 5)^12,28,30,31^. As in Unit 1, the ripple sets in Unit 2 prograde and retrograde up-section, which again form zig-zag stratigraphic patterns in 2D vertical sections (Figs. 3, 4; Supplementary Videos S1 and S2). A common feature of the Unit 1 and 2 travertine, which further accentuates the travertine crystal growth ripple morphology, is the deposition of siliciclastic sand grains within eddies on the lee slope of ripple sets (Figs. 4A, B, D, 5A), called *lee sands*^12^ (Fig. 3A–C). These lag deposits contain a minor component of an assortment of fine- to coarse-grained and angular to rounded siliciclastic sands^12^. MicroCT imaging prior to hand-sample cutting indicates that these sands were originally densely packed on the lee side of each ripple prior to being washed out and plucked during sample preparation, leaving behind void spaces (Fig. 4A, B)^12^.

### Quantitative characterization of travertine crystal-growth ripples

**Figure 6:**
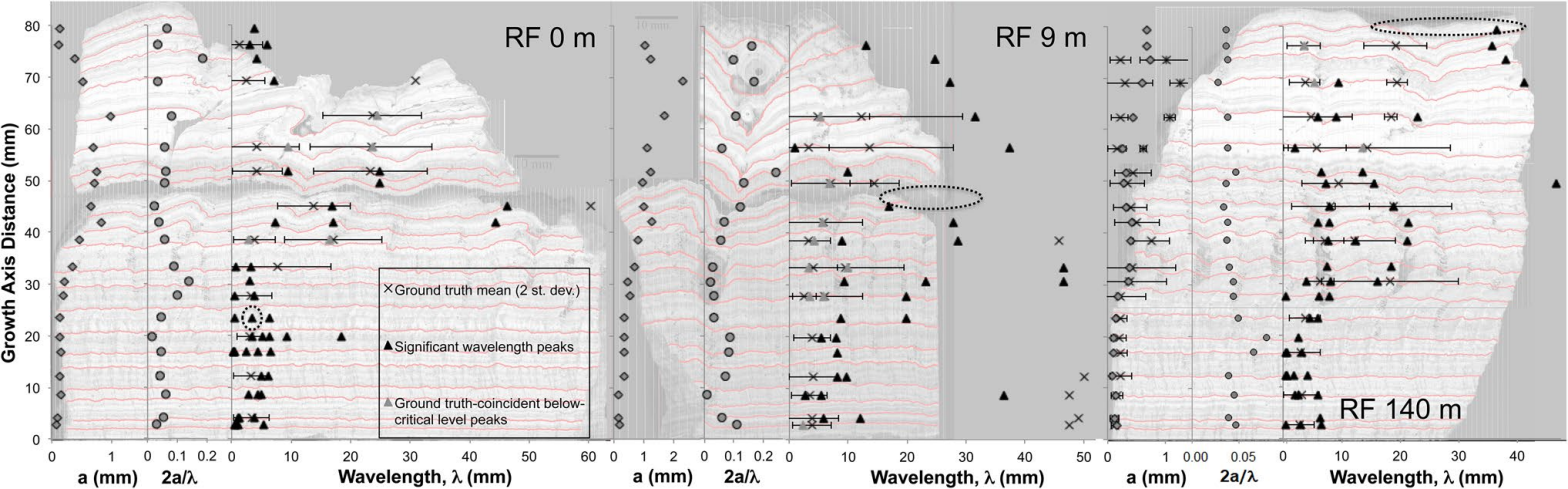
Travertine crystal growth ripple morphology cross-section measurements and characterization within stratigraphic cross sections of the Anio Novus aqueduct deposits at Roma Vecchia (Fig. 1B). The thickness of vertical travertine accumulation from the time-zero surface (t_0_) is plotted on the y-axis. Aqueduct water low direction is from left to right in all three images. Travertine wavelength (λ) is shown on the x-axis, as well as uncertainty bars corresponding to two standard deviations. Left: Sample RF 0 m (number of samples analysed (n) = 21). Center: Sample RF 9 m (n = 20). Right: RF 140 m (n = 21). Data is presented in Supplementary Data Files 1–3.

Our approach to quantifying up-section stratigraphic variation in travertine crystal growth ripple geomorphology for the 0, 9 and 140 m samples collected at Roma Vecchia (Fig. 1B) are shown in Fig. 6. The digital horizons analysed by Fourier transform are shown by red line tracings overlaid on the hand-sample images (Fig. 6). There is generally good agreement between the shortest significant wavelengths identified by the Fourier transform (shown as black triangles) and ground-truthed wavelengths (shown by crosses). After initial establishment of the travertine crystal growth ripples at the base of Unit 1 (Fig. 4A, B), a cyclic stratigraphic succession of ripple wavelength and amplitude occurs from increasing, to decreasing and increasing again.

The calculation of ${Re}_{c}^{*}$ from mean travertine crystal growth ripple morphology wavelength, water temperature and kinematic viscosity and shear velocity has been completed at the three different sites along the flow path of the Anio Novus (Fig. 1A). Full measurements of travertine crystal growth ripple characteristics and results are provided in the Supplementary Information (S3.2).

### Calculation of critical shear reynolds number from aqueduct travertine crystal-growth ripples

*Shear velocity, *${u}^{*}$*, and *${Re}_{c}^{*}$ (Supplementary Table S2) *were computed using Eqs.* (1) and (3),* respectively.* In fluid-sediment systems, the boundary-averaged shear velocity (${u}^{*}$) is a means of expressing the boundary-averaged shear stress in units of velocity. ${u}^{*}$ can be determined from estimates of aqueduct longitudinal slope ($S$) and hydraulic radius (${R}_{h}$) by assuming uniform flow conditions, where^59^:

1 $${u}^{*}=\sqrt{\frac{{\tau }_{b}}{\rho }}=\sqrt{g{R}_{h}S}$$

where ${\tau }_{b}$ is the boundary-averaged shear stress, $\rho$ is the mass density of water, $g$ is the acceleration of gravity, and $S$ is the aqueduct floor slope (gradient). Equation (1) for shear velocity ${u}^{*}$ is based on the assumption of uniform flow and accounts, through the use of the hydraulic radius, for the irregular (associated with travertine deposition) and taller-than-wide shape of the wetted boundaries. Furthermore, it provides a value averaged over the whole wetted boundary. The present study has therefore adopted the assumption of uniform flow and based hydraulic computations on the geometric property of aqueduct channel hydraulic radius, defined as the ratio of flow area to wetted perimeter. The hydraulic radius ${R}_{h}$ is given by

2 $${R}_{h}=\frac{A}{P}$$

where $A$ is the flow area and $P$ is the wetted perimeter, which were estimated based on the travertine deposits on the floor and side walls of the cross section (e.g. Supplementary Fig. S12).

Investigations on a wide variety of mature convective heat/mass transfer crystalline morphologies have consistently found that ripple wavelength scales with the ratio between the water kinematic viscosity and the shear velocity (i.e., the thickness of the viscous sublayer). Furthermore, the shear-velocity based (or friction-based) Reynolds number ${\mathrm{Re}}^{*}$ has a constant critical value for the formation of different types of mass transfer morphologies^41,60–62^. This critical shear velocity-based Reynolds number ${\mathrm{Re}}_{c}^{*}$ is calculated as:

3 $${\mathrm{Re}}_{c}^{*}=\frac{{u}^{*}\overline{\lambda }}{\nu }$$

where $\overline{\lambda }$ is the boundary-averaged ripple wavelength and $\nu$ is the kinematic viscosity of water, which is a function of water temperature. Curl^57^ derived the following relationship between ${u}^{*}$, the mean (cross-sectionally-averaged) flow velocity ($\overline{u }$) and the Sauter mean of the measured ripple wavelengths (${\overline{\lambda }}_{32}$) for dissolution morphologies formed in a circular conduit:

4 $$\bar{u} = u^{*} \left( {2.5\left\lceil {\ln \left\{ {\frac{D}{{2\bar{\lambda }_{{32}} }}} \right\} - \frac{3}{2}} \right\rceil + B_{L} } \right)$$

where $D$ is the diameter of the conduit and ${B}_{L}$ is Prandtl's bed-roughness constant. ${B}_{L}$ is a constant for dissolution morphologies of a particular shape and ${\mathrm{Re}}_{c}^{*}$, once they have reached equilibrium with an imposed mean velocity $\overline{u }$ ^57,60^.

### Derivation of morphology roughness-flow relationships

We have modified Eq. (4) for use in the taller-than-wide, rectangular aqueduct channel through inclusion of the hydraulic radius (defined in Eq. 2), knowing that, for a circular conduit, ${R}_{h}=D/4$:

5 $$\bar{u} = u^{*} \left( {2.5\left\lceil {\ln \left\{ {\frac{{2R_{h} }}{{\bar{\lambda }_{{32}} }}} \right\} - \frac{3}{2}} \right\rceil + B_{L} } \right)$$

Substituting u* from Eq. (1) into Eq. (5) gives,

6 $$\bar{u} = \sqrt {gR_{h} S} \left( {2.5\left\lceil {\ln \left\{ {\frac{{2R_{h} }}{{\bar{\lambda }_{{32}} }}} \right\} - \frac{3}{2}} \right\rceil + B_{L} } \right)$$

Temperature determines the density and kinematic viscosity of water flows^63^, which in turn affects the flow depth and ${R}_{h}$. Through its incorporation of ${R}_{h}$, Eq. (6) thus negates the need to estimate the temperature of the flow that formed the travertine crystal growth ripples to estimate $\overline{u }$. If the wetted perimeter *P* and flow area *A* can be measured or estimated from the surviving travertine stratigraphy, then ${R}_{h}$ can be estimated from Eq. (2) and substituted into Eq. (6) to calculate $\overline{u }$. Then an estimation of flow rate, *Q*, can made using the following continuity equation:

7 $$Q=A\overline{u }$$

## Discussion

### Travertine crystal growth ripples

The stoss, crest, lee, and trough geomorphology of the Anio Novus travertine crystal growth ripples are consistently observed in 3D bedding surfaces (Figs. 2C, 3A–C, 5B, C) and in vertical 2D sections of hand samples and in 2D thin section (Fig. 4). While the complex physical, chemical, and biological processes controlling the travertine crystal growth ripples are distinctly different from those controlling sediment transport ripples^28,32^ and solution ripples^42^, the basic ripple nomenclature established in these previous studies provides an important comparative context for describing the travertine crystal growth ripple moprhologies^28,30,31^ (Figs. 3A–C, 4, 5). Of particular importance is that, in addition to their formation on the channel floor, the travertine crystal growth ripple morphologies of the Anio Novus also formed horizontally on the vertical surfaces of the aqueduct channel walls, such as those observed at Empiglione Bridge (Fig. 2C). This evidence indicates that the Anio Novus travertine crystal growth ripple morphologies are independent of gravitational forces (Fig. 5B, C). Similarly, non-gravity dependent flute, scallop, and ripple morphologies have been observed to form on the vertical walls and ceilings of ice and dissolving cave systems^34,35,37,38,42^. Conversely, all ripples formed from the downstream transport of suspended sediments are gravity dependent^28–31,40^, drawing another fundamental distinction with the Anio Novus aqueduct travertine crystal growth ripples.

Another previously well-studied class of gravity dependent heat/mass transport morphologies in travertine, called *microterracettes*, form in many different earth surface (i.e., lakes, rivers, cold- and hot-springs) and subsurface (e.g., caves, fractures) environments^2,3,42^. Each microterracette is structurally composed of a terraced pond and a lip, which create a gravity-driven cascading downstream sequence^2,3,64^ (Fig. 4D). Travertine microterracette morphologies are therefore geomorphologically distinct from the Anio Novus travertine crystal growth ripples (Fig. 5A–D) and are representative of deposition in fundamentally different gravity-independent regimes with respect to overall hydraulics, geochemistry, and biological activity^2,28,43,44,65,66^.

Valuable insights are provided via direct comparison of the Anio Novus travertine crystal growth ripple morphology with both modern and ancient Distal-Slope Facies travertine microterracettes deposited at Mammoth Hot Springs in Yellowstone National Park and in nearby Gardiner, Montana^2,64,67,68^. The Anio Novus travertine crystal growth ripples, which are slightly younger than the 38–52 CE age of the aqueduct itself^12^, were precipitated from the following gravity-independent aqueous conditions: (1) temperature = 6°–13.5 °C, pH = 7.8–8.4, and saturated with respect to carbonate mineral precipipation^53,69,70^; and (2) water depth = 1–2 m, flow velocity = 0.8–1.8 m/s, and highly turbulent confined channel flow^12,23,24^ (Fig. 5, Supplementary Table S3, Supplementary Figures S5, S13). In contrast, the Mammoth-Gardiner Distal-Slope Facies travertine microterracettes, which are modern to recent (0–8000 years old) at Mammoth and Pleistocene (19,500–38,700 years old) in the Gardiner quarries^2^, were precipitated under the following gravity-dependent, unconfined, nearly laminar sheet-flow aqueous conditions: (1) temperature = 28–44 °C; (2) pH = 7.3–8.1; (3) supersaturation (Omega) = 2–5; (4) water depth = 1–3 cm; and (6) flow velocity =  < 0.01–0.1 m/s^2,44,64,68,71^.

These comparisons reveal that the aqueous temperatures and chemistries are comparable between the formation of the Anio Novus travertine crystal growth ripples and the Mammoth-Gardiner travertine microterracettes, respectively. Furthermore, the mineralogy and crystalline structure of travertine crystal growth ripples and microterracettes are generally comparable. The Anio Novus travertine crystal growth ripples are composed of small 1–3 µm-diameter euhedral calcite crystals that form larger 100–500 µm-tall shrub-like aggregates^12^ (Fig. 4C, D). The Distal-Slope Facies microterracettes are composed of 1–3 µm-diameter euhedral calcite crystals that form 100 µm-tall shrub-like crystalline aggregates^2,64,68^. However, in stark contrast, there are orders-of-magnitude differences in the contextual flow regimes from which the Anio Novus travertine crystal growth ripples and Mammoth-Gardiner microterracettes were deposited, respectively.

### Hydraulic reconstructions from travertine crystal growth ripples

As described in the previous section, the travertine crystal growth ripples formed within the channel of the Anio Novus aqueduct are generally similar in 2D vertical section morphology to well-studied ripples formed during the downstream hydraulic transport of sedimentary grains (Fig. 5)^28^. This is despite having been formed under very different physical, chemical, and biological environmental conditions^2,3,28,31^, which is reflected in the morphology observed on 3D surfaces (Fig. 5). Importantly, Hanratty^33^ formulated a fluid mechanical theoretical framework for the development of instabilities on surfaces that are eroding, dissolving, or being precipitated. Results indicate that wave-induced turbulent flow is equally influential during convective diffusion controlling travertine CaCO_3_ crystal precipitation, as well as downstream sediment transport. Therefore, the hydraulic history of open-channel turbulent flow can be equally reliably reconstructed from both travertine crystal growth ripples and sediment transport ripple morphologies^33^.

To compare aqueduct travertine crystal growth ripples characteristics with those of other heat/mass transfer crystalline morphologies (Supplementary Information S4.1), Supplementary Table S1 summarizes estimates of the ripple wavelengths at Empiglione Bridge (“latest flow”), Galleria Egidio (“early flow”), and Roma Vecchia (“latest flow”). Supplementary Table S2 establishes the shear velocity, Reynolds number and other flow properties from the data in Supplementary Table S1. The aim is to evaluate a possible relation between ripple wavelength along the flow direction and other flow properties. This was completed on the travertine crystal growth ripples observed in 2D channel sections from the three sites along the Anio Novus. Requirements included having enough travertine preserved to determine the hydraulic radius (${R}_{h}$), given by the ratio of flow area $A$ and wetted perimeter $P$ (Eq. 2), could be reconstructed and where the assumption of uniform flow is valid^23,24^.

**Figure 7:**
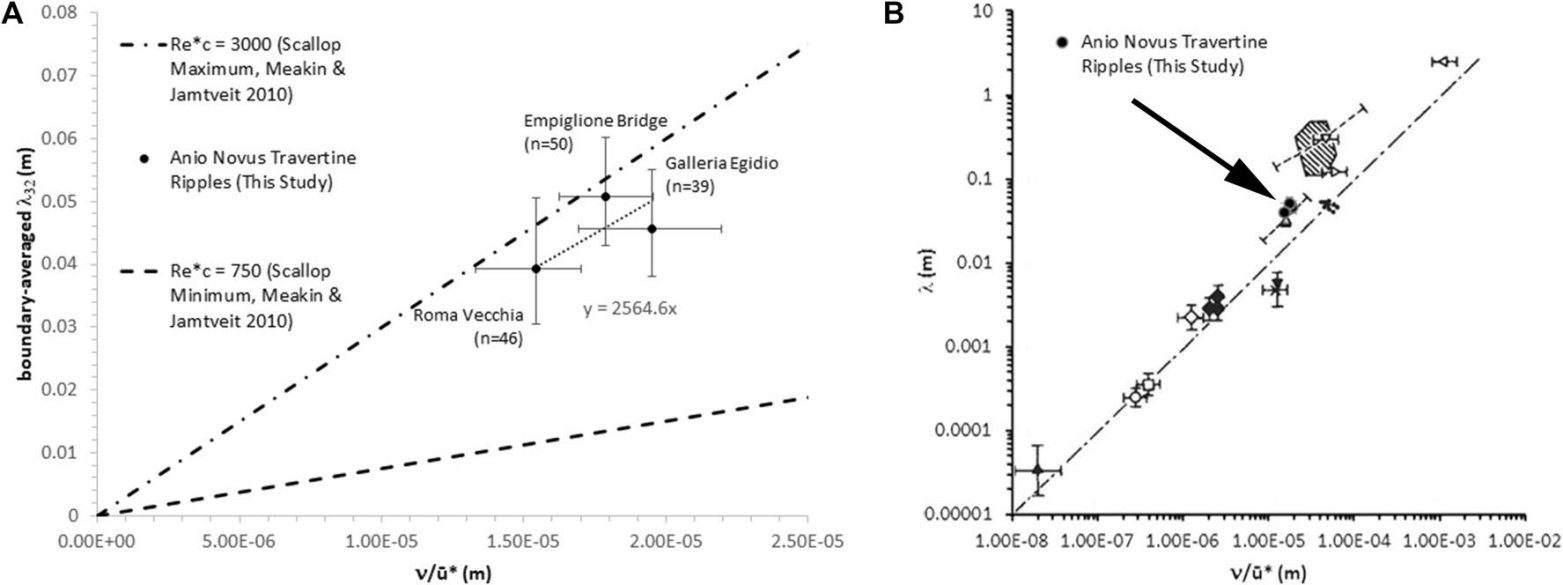
Covariation between ${{\varvec{u}}}^{\boldsymbol{*}}$ and ${\overline{{\varvec{\lambda}}} }_{32}$ (see Supplementary Table S2). (**A**) Data from this study falling within dashed lines representing Meakin and Jamtveit (2010)’s range for Re* of scallop formation. “n” refers to number of measurements from which ${\overline{{\varvec{\lambda}}} }_{32}$ was calculated. Dotted line is a linear regression with details shown on the chart. Uncertainty bars correspond to two standard deviations. (**B**) The same Anio Novus travertine crystal growth ripple data (from **A**) now plotted over Thomas^61^’ log–log plot of data from sediment transport ripples, solution ripples (scallops eroded on metal, limestone, bitumen and plaster surfaces) and ice ripples. Anio Novus travertine data plot amongst these other ripple and scallop data (used with permission).

Figure 7 plots $\nu$/${u}^{*}$ versus ${\overline{\lambda }}_{32}$ for the three sample sites along the Anio Novus flow path (Fig. 1A), all the sites available due to the limited survival and accessibility of archaeological remains. The wavelength for each of these 3 points is averaged from the wavelengths of 39–50 individual travertine crystal growth ripples at that location (Supplementary Information S5.3, Supplementary Figures S7-S11). Uncertainties and approximations also resulted from data collection challenges related to the evaluation of: (1) channel slope (Galleria Egidio) and wetted perimeter (Roma Vecchia); (2) the shear velocity estimate (carried out using a uniform-flow assumption and a boundary-averaged expression for the shear stress) and (3) the fluid viscosity (computed for constant water temperature, which is a reasonable approximation in the aqueduct based on the water temperature data at the source, Supplementary Information S5.4). Nevertheless, the critical shear Reynolds numbers for travertine crystal growth ripple formation for each site (Supplementary Table S2) and for the Anio Novus overall (c. 2565, obtained by linear regression between the sites, Fig. 7A) consistently fall within the range of previously measured dissolution and precipitation morphologies, such as 750 $\precsim {\mathrm{Re}}_{c}^{*}\precsim$ 3000^41,61,72,73^. The linear relationship suggested by the data shown in Fig. 7A. is in close agreement with past work on a large variety of morphologies (such as scallops and ripples, formed both by sediment grain deposition^32^ and crystalline precipitation/dissolution^42^), which found a linear relationship between these same properties of $\nu$/${u}^{*}$ and $\lambda$ (Fig. 7B). This suggests that, as in dissolution morphologies^42,72^, the ripple wavelength scales with the thickness of the viscous sublayer (δ), which is commonly estimated as 11.6 $\nu /{u}^{*}$^59^. In other words, if the thickness of the viscous sublayer increases, so does the ripple wavelength. More precise data are clearly needed, and additional sites within the Anio Novus deposits exhibiting travertine crystal growth ripples will be sought in future studies where uniform flow can be assumed and where slope, wetted perimeter, flow area and ripple wavelength can be measured. Nevertheless, for the first time in aqueduct travertine deposits, this shows that travertine crystal growth ripple wavelength varies due to changes in shear velocity and fluid viscosity (i.e., water temperature^63^) alone, in accord with Eq. (3).

Unlike dissolution morphologies such as scallops^42^, which dissolve in earlier formed bedrock, travertine crystal growth ripples deposit new layers on top of previous ripple sets, thus recording their history of formation. Therefore, changes in flow conditions and/or temperature over time can be traced upwards through changing travertine crystal growth ripple characteristics (illustrated by the red and green lines in Fig. 4, which provide 2D upward tracing of troughs in vertical section). It is shown above (Fig. 7) that the Anio Novus travertine crystal growth ripples are within the range of critical Re* for dissolution morphologies, suggesting that Curl’s Eq. (4), and hence our Eq. (6), are valid for estimating flow velocity from travertine crystal growth ripple wavelengths. Using Eqs. (6) and (7), flow velocity and rate for the three Anio Novus sites were calculated from travertine crystal growth ripple wavelength and other data in Supplementary Table S2. These results, shown in Supplementary Table S3, illustrate that the mean velocity $\overline{u }$ estimated from travertine crystal growth ripple wavelength is within 12–16% of that obtained using the uniform-flow Manning’s equation with an estimated Manning’s roughness coefficient (*n*) typical of a material with similar roughness to the aqueduct travertine. This confirms that Eqs. (4) and (6) are at least broadly valid for interpreting travertine crystal growth ripples. Further confirmation is provided by the travertine crystal growth ripples-flow relationships developed for sediment ripples by Van Rijn^74^ from the resistance equation, which make use of the ripple morphology height, $\Delta ,$ as well as the ripple wavelength (Supplementary Information, Section S4.3). Moreover, our modified Eq. (6) has the advantage of accounting for changes in travertine crystal growth ripple characteristics over time at a particular site, as well as at different sites along the flow path, permitting the reconstruction of the histories of flow velocities and rates throughout a Roman aqueduct network and in similar networks.

Travertine 2D cross-sections such as those of Units 1 and 2 from the aqueduct floor at Roma Vecchia (Fig. 4A, B) are ideal for such reconstructions, since the up-section changes in ripple wavelength can be traced over time through the stratigraphy. However, the travertine on the walls was too poorly preserved at this location to measure or estimate wetted perimeter, *P,* and flow area, *A*, for Units 1 and 2. This meant that hydraulic radius could not be estimated, which precluded the use of Eq. (6). Nevertheless, qualitative changes in flow velocity and rate during Units 1 and 2 can be interpreted. From Eq. (3), increases in travertine crystal growth ripple wavelength only result from decreases in shear velocity and/or increases in kinematic viscosity. Kinematic viscosity will vary with temperature^63^, but, as detailed in Supplementary Information S4.4, the limited possible range of temperatures means variation in viscosity would have been significantly smaller (+ 13/ − 10% of the representative 10 °C value, Supplementary Information S5.4) than the observed variation in wavelength at Roma Vecchia (+ /-70–150% of the mean). This means that the majority of observed changes in travertine crystal growth ripple wavelength in depositional Units 1 and 2 of Roma Vecchia travertine were due to changes in shear velocity (i.e., flow discharge) at the site rather than changes in temperature. Whereas changes in shear velocity would have been primarily due to changes in flow velocity rather than other factors (Supplementary Information S4.5). Hence, under near-constant kinematic viscosity, the mean wavelength increases observed in Anio Novus travertine are evidence of decreases in flow velocity and rate in the aqueduct, and vice versa. This is confirmed, theoretically and experimentally, for heat/mass transfer morphologies by many previous studies^75–80^.

As a result, qualitative changes in flow velocity and rate were reconstructed for the earliest periods of flow that formed the 8-cm basal of the deposit within the Anio Novus aqueduct channel at Roma Vecchia (Fig. 4A, B). This was completed using 2D stratigraphic up-section changes in the wavelength of the travertine crystal growth ripples (Fig. 6) deposited on the previously flat t_0_ channel mortar surface or on bed defects. After a period of travertine crystal growth ripple initiation (bottom half of Unit 1), flow rate then decreased markedly (third quarter of Unit 1), as suggested by the increase in ripple wavelength at RF 9 m and 140 m (Figs. 4A, B, 6). Flow rate then increased (top quarter of Unit 1), reducing ripple wavelengths, reaching a maximum at the Unit 1/Unit 2 interface. A second reduction in flow rate occurred in the bottom half of Unit 2, after which flow rate remained relatively constant (according to RF140 m). These reductions likely occurred during the first few centuries of the operation of the Anio Novus, i.e. 52 – c.250 CE, when water demand at Rome was near its peak^81^. This interpretation is discussed in more detail in Supplementary Information S4.6.

### Implications for reconstructions of climate and human activity

Possible drivers of these changes in flow rate in the Anio Novus include climate variability and human action or inaction. However, neither of these could have pushed the flow rate above the limit of 2 m^3^/s imposed by the hydraulic constraints and bottlenecks presented above^23^. Climate may have affected the flow in Anio Novus by changing the flow rate input at its source, the Aniene River, through changing rainfall amounts in the upstream catchment. This seems unlikely to have been significant, however, since the Aniene River could probably have always supplied more water to the aqueduct than it could carry (Supplementary Information, S4.7). Therefore, human manipulation of aqueduct flow was likely the major control of changes in flow within the Anio Novus rather than climatic variations.

There would have been two predominant forms of human action^21^: (1) intentional removal of water from the aqueduct, either legally sanctioned or fraudulent; and (2) maintenance (or lack thereof). It seems likely that Roman managers would have worked to maintain flow rate in the upstream reaches of the Anio Novus aqueduct as close to the carrying capacity of the channel as possible. Frontinus^21^ regards high flow rates in aqueducts as positive, given the ongoing high demand for water in the city, and laments the loss of water *en route*. Even if there were sluice gates at the aqueduct intake (a contested point^49,55^) it seems unlikely that these gates were used to reduce inflows significantly below the capacity of the channel. Diversionary channels to lower aqueducts, properties, and towns such as Tivoli, may have exerted some control on flow via the use of sluice gates or other means^21,49^. Closing of the sluice gates might also have been used as a means to temporarily impound water within the channel itself^82^. Lack of maintenance^20^, would have allowed travertine deposition to constrict the channel flow, while its weight may have caused leakage from structural cracks. Such cracks could also have resulted from weathering or earthquakes. Constriction and cracking would both have reduced flow within the aqueduct. Travertine deposits 10 s of cm-thick on the floors, walls and ceiling of the aqueduct channel internal perimeter in many areas of the Anio Novus^49^, at sites such as Osteriola, indicate that regular maintenance was neglected, at least towards the operational demise of the Anio Novus aqueduct.

## Conclusions

Travertine crystal growth ripples have been utilized in this study to reconstruct the early hydraulic history of the Anio Novus, the largest and farthest reaching of ancient Rome’s 11 aqueducts. Travertine crystallizes from complexly physical, chemical, and biological processes that operate fundamentally differently from those controlling sandstone ripple mark formation. Yet despite these mechanistic distinctions, the hydraulic history of gravity-driven turbulent flow can be reliably reconstructed from travertine crystal growth ripples as they are from sediment transport ripples. As a result, the hydraulic significance of the amplitude and wavelength of travertine crystal growth ripples preserved within aqueduct travertine have been used to reconstruct the hydraulic history of the Anio Novus. This included petrographic characterization and measurement of travertine crystal growth ripples deposited on the channel floor, walls, and roof of the Anio Novus aqueduct, arguably the most significant of the 11 aqueducts that supplied water to Imperial Rome. Of particular importance is that the critical shear Reynolds number of the travertine crystal growth ripples are within the range of hydraulic parameters previously observed to form ripples in multiple other analogous natural and manmade environments. These similarities have been used to further improve upon our previous uniform flow estimates of velocity and discharge for the Anio Novus aqueduct. These relationships establish travertine crystal growth ripple wavelength, defined as the characteristic length of the shear Reynolds number, as a fundamental parameter required to confidently reconstruct maintenance and management of the Anio Novus aqueduct.

## Supplementary Information

Supplementary Information 1.

Supplementary Information 2.

Supplementary Information 3.

Supplementary Information 4.

Supplementary Video 1.

Supplementary Video 2.

## Data Availability

Raw unprocessed data and images for Main Figures and SI Figures will be made available in the original source format on the University of Illinois cloud data base.
