# Supplementary material for: Travertine crystal growth ripples record the hydraulic history of ancient Rome’s Anio Novus aqueduct
Source: Sci Rep. 2022 Jan 24;12:1239. doi: 10.1038/s41598-022-05158-2 (PMC8786873; doi:10.1038/s41598-022-05158-2)
Supplement: Supplementary file 4 — Supplementary Information 4. [file 41598_2022_5158_MOESM4_ESM.pdf]

Supplementary Information for "Travertine Ripple Marks Record the Hydrology of Ancient Rome's Anio Novus Aqueduct"

Duncan Keenan-Jones[a], Davide Motta[b], Marcelo H. Garcia[c], Ryan K. Shosted[d], Mayandi Sivaguru[e], Mauricio Perillo[f], and Bruce W. Fouke[g]

[a]School of Historical and Philosophical Inquiry, The University of Queensland, St Lucia QLD 4072, Australia d.keenanjones@uq.edu.au (corresponding author)

[b]Northumbria University, Wynne Jones Building, Newcastle upon Tyne, NE1 8ST, United Kingdom, davide.motta@northumbria.ac.uk

[c]Ven Te Chow Hydrosystems Laboratory, Department of Civil and Environmental Engineering, University of Illinois at Urbana-Champaign, 205 North Mathews Avenue, Urbana, IL, 61801, USA, mhgarcia@illinois.edu

[d]Department of Linguistics, University of Illinois at Urbana-Champaign, Champaign, USA, rshosted@illinois.edu 707 S Mathews Ave., Urbana, Illinois, USA, rshosted@illinois.edu

[e] Carl R. Woese Institute for Genomic Biology, University of Illinois at Urbana-Champaign, Urbana, IL, USA and Carl Zeiss Labs@Location Partner

[f]Department of Geology, University of Illinois at Urbana-Champaign, 1301. W. Green St., Urbana, IL 61801, USA. Present Address: ExxonMobil Upstream Business Development, mauricio.m.perillo@exxonmobil.com.

[g]Department of Geology, University of Illinois at Urbana-Champaign, 1301. W. Green St., Urbana, IL 61801, USA. Department of Evolution, Ecology, and Behavior, University of Illinois at Urbana-Champaign, Urbana, 505 S. Goodwin Ave., Urbana, IL, USA. Carl R. Woese Institute for Genomic Biology, University of Illinois at Urbana-Champaign, 1206 W. Gregory Drive, Urbana, IL 61801, USA. fouke@illinois.edu

S1 Introduction

Examples of heat/mass transfer crystalline bedforms include: heat/mass transfer crystalline bedforms include: (i) ripples on ice surfaces formed by the melting and freezing of water[1,2]; (ii) flutes formed by melting on the underside of river ice[3]; (iii) cave limestone (speleothem) crenulations[4]; (iv) polygonal scallops and transverse flutes formed in karsted cave systems[5,6]; (v) "waves" in calcium carbonate pipe scale[7]; and (vi) travertine terraces found in rivers[8], caves, and particularly hot springs[9].

S2 Regional Setting

The height of the Anio Novus was less important for supply to the city itself, since multiple aqueducts were high enough to supply almost all of the city[10], even the areas across the Tiber[11]. In the more elevated Aniene valley, however, its greater elevation was vital to feed diversionary channels at several locations that could “top up” the three lower aqueducts, and to supply elite villas located above the other aqueducts, such as the villa of “Sette Bassi” at Roma Vecchia[12]. The function of the channel bifurcation in the Anio Novus upstream of Tivoli (Fig. 1) remains debated, but was likely constructed to supply properties in and around Tivoli and/or to facilitate aqueduct maintenance in this region[13].

S3 Results

S3.1 Heat/Mass Transfer Crystalline Bedforms

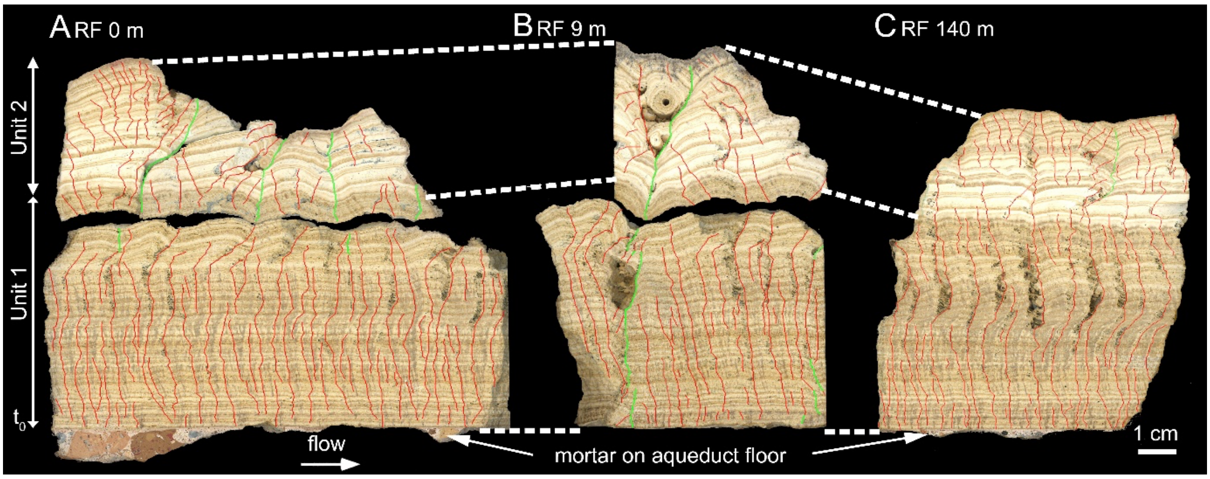

Supplementary Figure S1 Stratigraphic cross-sections oriented parallel to the upstream-downstream flow direction of the ripple-marked travertine deposited within the Anio Novus aqueduct at Roma Vecchia (Fig. 1). A. Travertine collected from the farthest upstream RF 0 m site. B. Travertine collected from the intermediate RF 9 m site. C. Travertine collected from the downstream RF 140 m site. Red lines (mm-scale ripples) and green lines (cm-scale ripples) trace the vertical growth trajectory (*instability*) of ripple troughs during deposition. Stratigraphic correlation of the time-

zero (t_0) surface (contact with the underlying mortar on the aqueduct floor), the top surface of Unit 1, and the top surface of Unit 2 are indicated with white dashed lines.

Linguoid bedforms transition into sinuous bedforms within Unit 1 and the base of Unit 2, which eventually become irregular closely packed hummocky ripples toward the top of Unit 2 (Fig. 3, Supplementary Figs. S1-2, Supplementary Videos S1 and S2). The ripple bedforms at Galleria Egidio also exhibit this Unit 1 and base of Unit 2 transition from linguoid to sinuous (Supplementary Fig. S7), while the bedforms at Empiglione Bridge exhibit only Unit 1 sinuous ripples (Fig. 2C).

The quantitative analyses presented in the following have been ground-truthed with visual analyses of hand sample cross-sections (Supplementary Fig. S1). Ripples that formed within the Anio Novus travertine occur in multiple, superimposed scales and systematically vary up-section (i.e., with increasing vertical distance from t_0 surface) in three-dimensional (3D) shape, wavelength, amplitude and steepness. The ripples also generally have a common vertical growth trajectory of slight migration upstream or downstream (*instability*), as shown by the red and green lines in Supplementary Figure S1. In the stratigraphically lowest (oldest) part of each travertine deposit, localized variation in these characteristics is dependent on the size and position of surface irregularities (defects) in the t_0 substrate. Stratigraphically above this, successive ripple layers accumulate in both vertical (aggradational) and lateral (progradational) geometries at all three sample sites (RF 0 m, 9 m, and 140 m; Fig. 1), with ripple wavelength, asymmetry and especially amplitude increasing overall up section. The multiple superimposed scales of the ripple-marked travertine layers are similar to the superposition of ripples on larger bedforms in other natural sediment-transport systems [14]. The vertical growth trajectory of the largest ripple scale at the top of the section is mostly consistent within each horizon and between samples. Mm-scale ripples (red lines) are superimposed on

the larger cm-scale ripples (green lines) and the mm-scale ripples diverge over time as asymmetry increases (Supplementary Fig. S1).

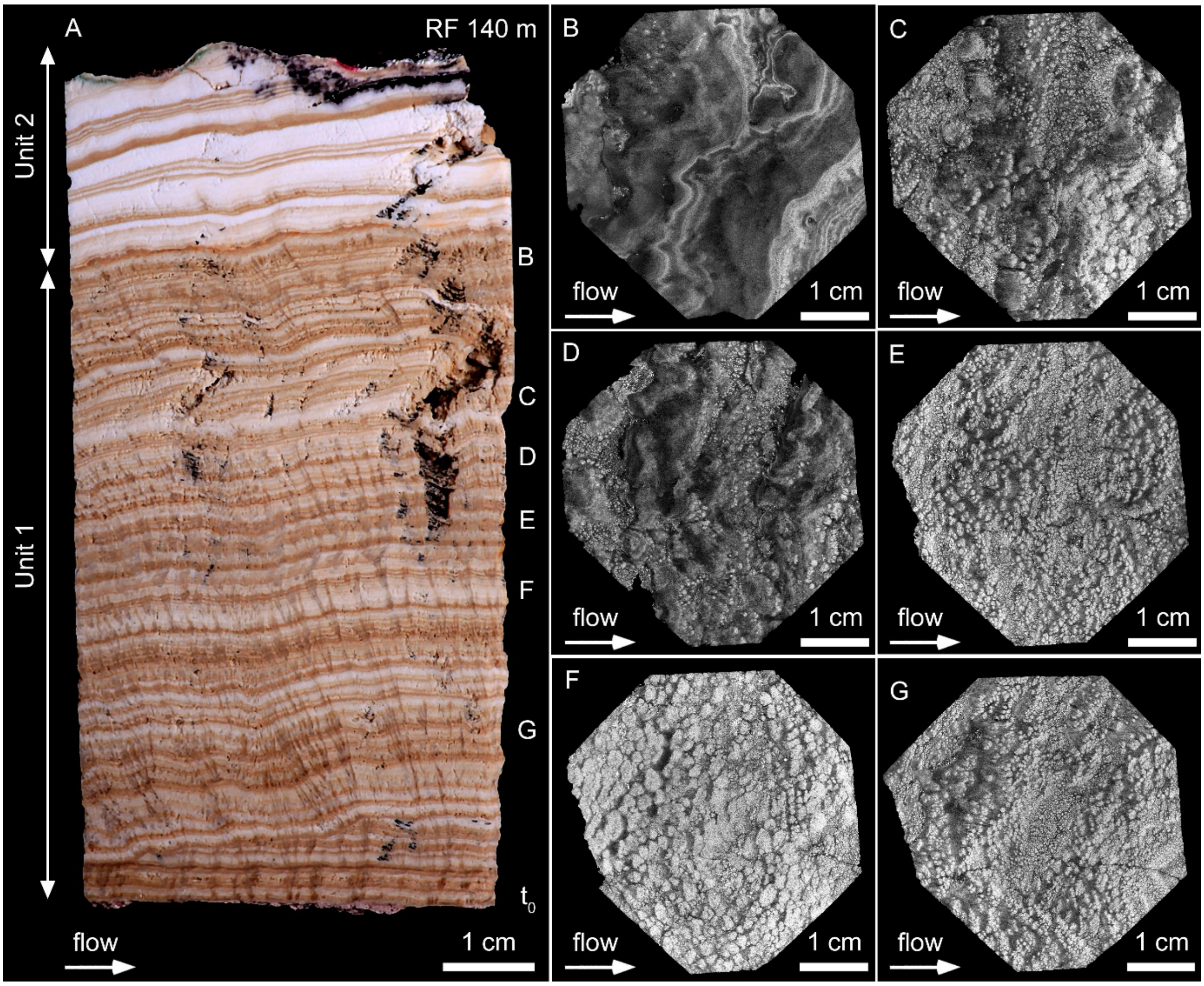

Supplementary Figure S2 Hand sample vertical cross-section photograph and virtual horizontal microcomputed tomography (microCT) cross sections of the ripple-marked travertine bedforms deposited at the farthest downsteam RF 140 m sample site within the Anio Novus aqueduct at Roma Vecchia (Fig. 1). The stratigraphic position of each virtual microCT cross section (B-G) is shown in the hand sample image A. This series of virtual cross sections illustrates the vertical stratigraphic succession of bedforms within Units 1 and 2, changing from low- to high-amplitude linguoid ripple crests in E, F, and G, to sinuous ripples in C and D and finally becoming hummocky ripples in B at the top of the stratigraphic section.

Initial irregularities have a strong and persistent effect on ripple development. Ripples of larger amplitude and wavelength have initially grown up from visible protrusions (defects) from the normally smooth channel floor. An example is the ripple in Supplementary Figure 1B between the upstream portion of the RF 9 m sample and the left-most green trough line extending from the bottom to the top of the deposit. This ripple has the greatest amplitude and asymmetry, as well as more downstream-transported SiO_2 lee sands, than any others observed at Roma Vecchia, which is manifested throughout the entire vertical stratigraphic sequence of the rippled travertine sample (Supplementary Fig. 1). A ripple of seemingly longer wavelength (almost as long as the sample itself), but smaller amplitude, is visible immediately downstream. Another example is the ripple behaviour created by the slope of the unconformity at RF 140 m, which is distributed throughout the entire stratigraphic section (Supplementary Fig. 1A), with a particularly large and steep lee face of the ripples.

Downstream instability (i.e., migration of troughs downstream over time with up-section accumulation) at the largest ripple scale generally occurs when wavelength is increasing, and upstream when the large-scale wavelength is decreasing (Fig. 6). RF 140 m is a particularly clear example of different wavelength scales migrating in concert with each other and with the instability. In this sample, the two largest wavelength scales and the amplitude increase when the instability at the largest scale is in the downstream direction and decrease when the instability is upstream.

S3.2 Quantitative Characterization of Travertine Ripples

At times during the quantitative characterization of the up-section variation of the travertine ripples at Roma Vecchia (Fig. 6), the chosen confidence interval excluded some spectral peaks that corresponded to wavelengths clearly visible during ground truthing ("false negatives" shown in Fig. 6 as grey triangles). Amplitude tends to move in the same direction as wavelength (i.e., as amplitude increases, wavelength does too, and vice versa). The changes in wavelength

are of greater magnitude than those in amplitude, however, steepness (2a/λ) develops in the opposite direction to wavelength. In general, asymmetric ripples, with long, gentle convex stoss slopes and short, steep lee slopes, are more common where wavelength is systematically increasing (Fig. 6).

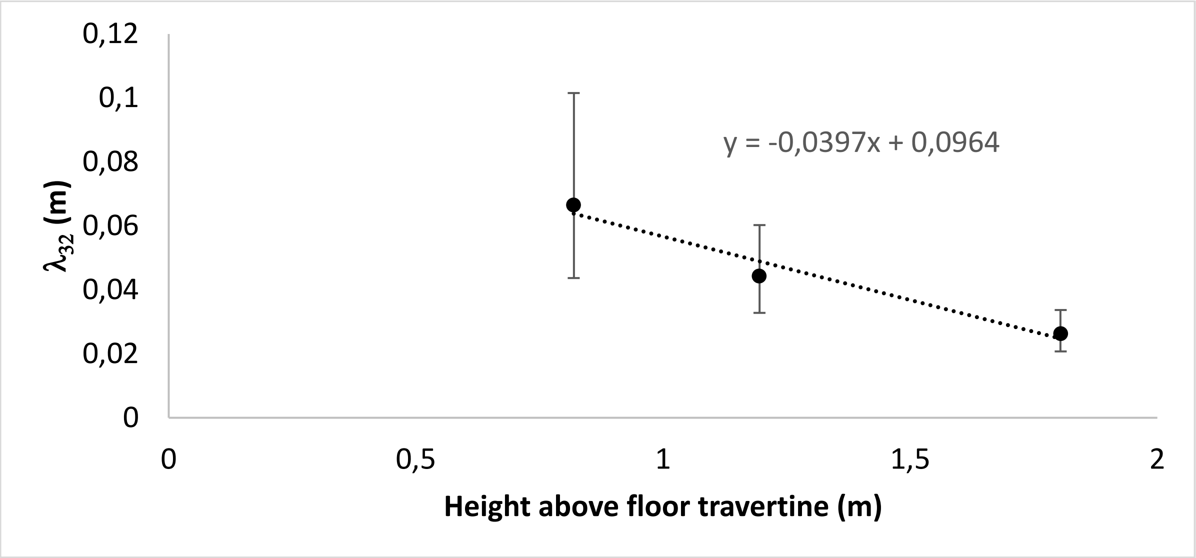

Supplementary Figure S3 Sauter mean of wavelengths (λ_{32}) at measured heights above the channel floor at Empiglione Bridge. Dotted line is a linear regression with details shown on the chart. Uncertainty bars are 2 standard deviations.

Where it could be measured on the wall and vault at Empiglione Bridge, the mean wavelength at each point decreases with increasing height (y) above the floor (Supplementary Fig. S3). It is difficult to tell whether the relationship is linear, exponential etc. from 3 points. From the non-boundary averaged versions of Equations 1 and 3, we would expect shear velocity and stress to be inversely proportional to wavelength. Shear stress decreases linearly with increasing y under uniform flow, however . This is the same as wavelength, rather than being inverse. This finding needs checking on more, and better preserved, rippled channels. If it is found to be generally true, then it deserves further investigation.

Site	Strati-graphic position	Location in the channel cross section and corresponding Sauter mean ripple wavelength (λ_{32})				Boundary-averaged Sauter mean of all wavelengths at site ($\bar{\lambda}_{32}$)
Empiglione Bridge	Latest flow	Vault surface (1.9 m above floor, n=24)	Upper wall surface (1.3 m above floor, n=15)	Middle wall surface (0.9 m above floor, n=9)	Loose fragment cross-section (position unknown, n=2)	51 +9/-8
		26 +7/-6	44 +16/-12	67 +35/-23	26 +13/–9	
Galleria Egidio	Early flow	Floor surface (n=38)		Wall cross-section (0.3 m above floor, n=1)		46 +9/-8
		45 +9/-8		55		
Roma Vecchia	Latest flow	Floor surface (n=45)		Floor cross-section (n=1)		39 +11/-9
		39 +12/-9		35		

Supplementary Table S1. Estimation of Sauter mean ripple wavelengths (λ_{32}) at Empiglione Bridge ("latest flow"), Galleria Egidio ("early flow"), and Roma Vecchia ("latest flow"). All measurements in mm. Uncertainties calculated according to Springer and Hall [15], Equations 1 and 2. "n" refers to the number of measurements. Where n =1, no uncertainty is given.

Site	Empiglione Bridge	Galleria Egidio	Roma Vecchia
**Cross-Section Shape**	Rectangular & circular vault	Rectangular & circular vault	Rectangular & circular vault
**Slope, S (m/m)**	0.0014	0.00125 +/- 0.00025[13]	0.00255
**Flow Area, A (m^2)**	1.850	1.142	1.199 +/-0.245[16]
**Wetted Perimeter, P (m)**	4.730	3.160	4.196 +0.437/ -0.063
**Hydraulic Radius, R_h (m)**	0.391	0.362	0.286 +0.026/- 0.055
**Boundary-Averaged Shear Stress, τ_b (N/m^2)**	5.33	4.49 +/- 0.8	7.14 + 0.65/-1.37
**Shear Velocity, u^* (m/s)**	0.073	0.067 +/-0.006	0.085 +0.004/ -0.009
**Thickness of Viscous Sub-layer, δ ($=11.6\nu/u^*$) (m)**	2.1 +0.19/-0.19 x 10^{-4}	2.3 +0.29/-0.30 x 10^{-4}	1.8+0.18/-0.25 x 10^{-4}
**Sauter Mean Ripple Wavelength, $\bar{\lambda}_{32}$ (m)**	0.051 +0.009/-0.008	0.046 +0.009/-0.008	0.039 +0.011/-0.009
**Site average critical shear Reynolds number, Re_c^***	2842 +654/-570	2344 +565/-506	2544 +776/-667
**Anio Novus average critical shear Reynolds number, Re_c^***	2565 +/-304		

Supplementary Table S2: Computation of shear velocity u^* and shear Reynolds number Re_c^* at Galleria Egidio ("early flow"), Empiglione Bridge ("latest flow"), and Roma

Vecchia ("latest flow"). All uncertainties are 2 standard deviations unless noted. Uncertainties in the slope at Galleria Egidio and in the flow area and wetted perimeter at Roma Vecchia have been propagated as if they were 1 standard deviation. Anio Novus Re_c^* and its 2-standard-deviation uncertainty were determined using the LINEST function in Microsoft Excel.

The measured bedform heights (Supplementary Table S1) are all 30-40 times larger than the viscous sublayer (Supplementary Table S2) at the stratigraphic horizons and surfaces investigated, meaning that the bed is hydraulically rough.

Blumberg and Curl[17] estimate B_L as 9.4 for 3D dissolution bedforms ("scallops", analogous to linguoid precipitation bedforms, Fig. 3A) with an Re_c^* of 2200. The slightly higher Anio Novus Re_c^* of 2565 (Fig. 7) suggests a slightly lower B_L value, 9.0 +/- 1.5[Fig. 6 in 17], which has been used in calculations in the present study. Equation 6 is sensitive to the value of B_L, and would benefit from the experimental determination of B_L for aqueduct travertine bedforms.

Site	Empiglione Bridge	Galleria Egidio	Roma Vecchia
**Sauter Mean Ripple Wavelength, $\bar{\lambda}_{32}$ (m)**	0.051 +0.009/-0.008	0.046 +0.009/-0.008	0.039 +0.011/-0.009
B_L at Re_c^* = **2565**[Fig. 6 in 17]	9.0 +/- 1.5	9.0 +/- 1.5	9.0 +/- 1.5
**Cross-sectionally-Averaged Flow Velocity from bedforms via Eqn 6, $\bar{u}_b$ (m/s)**	0.88 +0.12/-0.11	0.81 +0.13/-0.13	1.0 +0.2/-0.2
**Cross-sectionally-Averaged Flow Velocity from assumed Manning's *n* = 0.019, $\bar{u}_n$ (m/s)**	1.05	0.95 +0.08/-0.09	1.2 + 0.07/-0.15
**Flow Velocity Percent Difference** $(\frac{\bar{u}_b - \bar{u}_n}{\bar{u}_n})$	-15.9%	-14.3%	-12.5%
**Flow Rate from Bedforms via Equation 7, Q_b (m³/s)**	1.6 +0.2/- 0.2	0.93 +0.15/-0.15	1.2 +0.4/-0.5
**Flow Rate from assumed Manning's *n* = 0.019 sm$^{-1/3}$, Q_n (m³/s)**	1.9	1.1 +0.1/-0.1	1.4 +0.4/-0.4
**Flow Rate Percent Difference** $(\frac{Q_b - Q_n}{Q_n})$	-15.9%	-14.3%	-12.5%

Supplementary Table S3. Anio Novus flow characteristics determined from bedform and channel geometry alone (Equations 6 and 7). All uncertainties are 2 standard deviations. Uncertainty in $\bar{u}_n$ at Empiglione Bridge is unknown since the uncertainty in slope, wetted perimeter and flow area, as well as the degree of deviation from perfect uniform flow, cannot be quantified with the data available.

S4 Discussion

S4.1 Heat/Mass Transfer Crystalline Bedforms

It has been demonstrated by previous studies that convective heat/mass transfer crystalline bedforms begin at points of surface roughness (defects) that locally affect flow and hence mass transfer[6,17–19], which is consistent with our observations of the rippled-marked aqueduct travertine at Roma Vecchia as outlined above. Crystalline bedforms precipitated from chemically supersaturated water could result from several different mechanisms that are capable of causing increased precipitation at or near these defect irregularities[20,21]. The first is driven by the reduction of the depth of the flow as it passes over a raised surface defect[9]. This shallowing causes increased CO_2 degassing and increased velocity due to the Bernoulli effect, which leads to thinning of the boundary layer and increased diffusion. Another mechanism is the deformation of concentration gradients in the viscous sublayer of the precipitating waters due to flow disruption caused by the surface defect. In Roman aqueducts such as the Anio Novus, great care was generally taken during Roman construction to smooth the mortar lining of the channel. As a result, the depth of flow within aqueduct channels was significantly larger than the height of any mortar surface defects and shallowing would have had a minimal effect. Therefore, the presence of well-preserved travertine ripples in the absence of significant shallowing suggests that the viscous sublayer was the most important hydraulic factor within Roman aqueducts. The development of mass and heat transfer bedforms parallel to the flow direction has been the focus of most studies. Downstream of the defect, there is a stagnant zone (due to eddies) with lower precipitation rates. Immediately downstream of the stagnant zone there is an increase in precipitation rate due to mixing[20], setting the stage for a series of upstream-to-downstream travertine bedforms.

Much less is known about the development of bedforms perpendicular to the flow direction and hence also about the conditions leading to the formation of the different planform geometries in heat/mass transfer bedforms. Blumberg and Curl[17] could only reproduce 3D

dissolution scallop bedforms in the laboratory (i.e., where each bedform varies in shape in all three dimensions). These scallops are analogous to linguoid and perhaps hummocky bedforms. Constant temperature and velocity, conjectured to be necessary[5], did not prove sufficient to produce 2D dissolution bedforms (flutes, where the cross-sections parallel to the flow of a single bedform are similar across the whole channel). The bedform developmental sequence observed in the Anio Novus differs significantly from the known pattern of current ripples in non-cohesive sediment[22–24]. The first two stages observed here (linguoid and sinuous) form in non-cohesive sediment under unidirectional current (such as rivers) that are a good analogue for the downstream flow prevailing in the aqueduct. The natural order, however, is normally reversed (i.e. sinuous then linguoid) since ripples in non-cohesive sediments show increasing three-dimensionality in vertical cross-section[22]. Hummocks form in non-cohesive sediments under the combined effect of unidirectional and short-period oscillatory currents, e.g. surface waves[23,25]. However, it is extremely unlikely, if not impossible, that oscillatory currents occurred in the flow of the Anio Novus.

Initially, the ripple wavelength is determined by the spacing of surface irregularities[18], resulting in significant localized variation. However, once flow properties come to dominate, the mature travertine ripples exhibit a more consistent wavelength, probably due to the onset of significant flow separation[3,17,26,27]. Once the steepness (equivalent to bedform height/wavelength) of the bedforms reaches a critical value, the flow downstream of the crest separates from the bed. As a result, the bedforms become[3], or can become[28] asymmetric with a gentle stoss slope and a steep lee slope. Such asymmetric bedforms are found in the Roma Vecchia deposits, but cannot be related to a steepness threshold (Fig. 6). Probably the flow-separated area, representing separation from the supply of fresh ions in the bulk flow, has a lower mass transfer (precipitation or dissolution) rate than the location downstream where the flow reattaches to the surface[18,29]. The largest bedforms would then starve smaller bedforms within their downstream separated flow and start to grow new ripple crests at or just

downstream of the point of reattachment. This mechanism has been demonstrated in the laboratory. Prefabricated 2D dissolution bedforms (flutes) of set wavelengths have been shown to change wavelength to reflect imposed velocities and Reynolds numbers[17]. Wavelength change occurred by changing the number of crests, where some crests were suppressed during wavelength increase, while smaller-scale features appearing on the scallop stoss-sides began to overprint the pattern, during wavelength decrease.

S4.2 Instability in Anio Novus Travertine

One bedform characteristic which could be used to reconstruct the flow conditions that formed the bedforms is instability. For crystallization from a laminar falling film, such as stalactites (Camporeale and Ridolfi, 2012a) and icicles[30–32], upstream instability is dominant, although earlier theoretical calculations predicted downstream instability in icicles[33], and the rate (or angle) of upstream instability is inversely proportional to Reynolds Number ($10^{-3} < Re < 10^{-1}$). Maximum mass transfer is generally on the stoss side of bedforms[17], probably because this is generally the location of flow reattachment. Instability in precipitation bedforms is thought to occur by adding to and extending crests upstream towards this location of maximum mass transfer[21], thus upstream instability should be the norm. Upstream instability would result in the fastest increase or decrease in wave amplitude. Under downstream instability, amplitude decrease would be much slower and increase practically impossible. Thus for ripples to form or grow, instability should be upstream. Hanratty's modelling and partial measurement of the phase difference between the location of maximum mass transfer and the crest is the only known previous work that considered precipitating bedforms under turbulent flow, i.e. a situation similar to that of the aqueduct.

Instability in Roma Vecchia travertine differs significantly from this previous research, however, and additional analyses would need to be completed to firmly establish these relationships. The loose correlation between (mature) wavelength and instability in the Roma

Vecchia deposits implies that upstream instability occurs when flow rate and Reynolds Number is increasing, i.e. the opposite relationship to that prevailing under the laminar falling films of stalactites. Roma Vecchia travertine also shows instability in both the upstream and downstream direction, which has not previously been observed in the same system[31,34]. The initial instability at Roma Vecchia is slightly upstream as Hanratty suggests that it should be. The upstream instability corresponding with amplitude growth in the bottom of Unit 1 and with amplitude reduction towards the top of Unit 1 both accord with Hanratty's predictions. Both in the middle of Unit 1 and in Unit 2 in the RF 9 m and RF 140 m samples, however, there is strong amplitude growth coupled with downstream instability, contrary to Hanratty's theory described above. The ripples in these two areas are strongly asymmetric, implying significant flow separation.

S4.3 Confirmation of Equation 6 using Bedform Roughness Height and Van Rijn's Relationships

A further means of assessing the validity of our Equation 6 is provided by the bedform-flow relationships developed for sediment ripples by Van Rijn[35] from the resistance equation, which make use of the bedform roughness height, Δ, as well as the bedform wavelength (through incorporation of steepness). Hydraulic rough flow occurs at all three Anio Novus sites (Supplementary Table S4), so the mean velocity $\bar{u}$ can be calculated from Van Rijn's Equation 7 as follows:

$$\bar{u} = u^{*}\left(2.5ln\left\{\frac{R_h}{k_c}\right\} + 6.23\right) = u^{*}\left(2.5ln\left\{\frac{R_h}{k_s+k_f}\right\} + 6.23\right) \qquad \text{(S1)}$$

where k_c is the equivalent roughness height, which is the sum of k_s the roughness component due to skin friction and k_f the roughness component due to the form drag associated with the bedforms[36]. k_s is a surface property that is essentially independent of bedform characteristics: in sediment transport in rivers it is solely a function of grain size[36]. It can be estimated for

aqueduct travertine by substituting an equivalent Manning's n for a bedform-free surface ($k_f = 0$) into the Strickler formula[36] as follows:

$$n = \frac{k_c^{\frac{1}{6}}}{8.1g^{\frac{1}{2}}} = \frac{(k_s + k_f)^{\frac{1}{6}}}{8.1g^{\frac{1}{2}}} \tag{S2}$$

Based on observations of aqueduct travertine in the Anio Novus, we have chosen Manning's n = 0.012, equivalent to a neat cement surface[37], for the surface roughness.

The most appropriate known expression for k_f is that of Van Rijn[35] for sediment ripples:

$$\frac{k_f}{\Delta} = 1.1\left(1 - e^{-25\frac{\Delta}{\lambda}}\right) \tag{S3}$$

Details and results of these calculations are given in Supplementary Table S4. The mean velocity $\bar{u}$ calculated from Equation S1 is consistently larger, although by only 20-36%, than the values obtained from Equation 6 at the three Anio Novus sites, providing further confirmation of the validity and results of Equation 6. Future work is needed to better estimate k_s and k_f for travertine to increase the accuracy of Van Rijn's Equation when estimating mean velocity; in the meantime, Equation 6 appears to be the preferable equation because it only relies on ripple wavelength, which can be more readily and univocally determined from travertine ripple profiles.

Site	Empiglione Bridge	Galleria Egidio	Roma Vecchia
**Equivalent Roughness Height due to Skin Friction, k_s (m)**	0.00080 +/- 0.00040	0.00080 +/- 0.00040	0.00080 +/- 0.00040
**Arithmetic Mean Ripple Wavelength, λ (m, cross-section measurements only)**	0.037 +/- 0.016	0.055 +-0.023	0.035 +-0.015
**Arithmetic Mean Ripple Height, Δ (m, cross-section measurements only)**	0.0084 +/-0.0018	0.0065 +/-0.0014	0.0083 +/-0.0018
**Steepness (=Δ/λ)**	0.23 +/- 0.11	0.12+/-0.06	0.23+/-0.11
**Equivalent Roughness Height due to Form Drag, k_f (m)**	0.0092 +/- 0.0249	0.0068 +/- 0.0096	0.0091 +/-0.0255
**Combined Equivalent Roughness Height, k_c (m)**	0.010 +/-0.0249	0.0076 +/- 0.0097	0.0099 +/- 0.0258
**Re (Reynolds Number =$u^* k_c/\nu$)**	560 +/- 1390	390 +/- 498	640+/- 1650
**Is flow hydraulicly rough (Re > 70)[35]?**	Yes	Yes	Yes
**Cross-sectionally-Averaged Flow Velocity from bedforms via Equation S1, u_c (m/s)**	1.1 +0.5/-0.5	1.1 +0.2/-0.2	1.3 +0.6/-0.6
**Cross-sectionally-Averaged Flow Velocity via Equation 6, u_b (m/s, using cross-section measurements only)**	0.88 +0.12/-0.11	0.81 +0.13/-0.13	1.0 +0.2/-0.2
**Flow Velocity Percent Difference** $(\frac{u_c - u_b}{u_b})$	26%	36%	20%

Supplementary Table S4. Mean flow velocity calculated using Equations S1-S3. Ripple height measurements were available only from the travertine sample cross-sections, so

only these Δ and λ measurments were used in Equations S1 – S3. The arithmetic mean of Δ and λ was used, following the practice of the experimental data used by Van Rijn to develop Equation S3[e.g. 38]. The hydraulic radius, R_h, and shear velocity, u^*, used were those in Supplementary Table S2.

S4.4 Temperature Variation in the Anio Novus

Heating as the water descended more than 300 m[13] in elevation along the entire flow path of the Anio Novus was minimal. Frontinus describes (Chapter 91.5) the refreshing coolness of aqueduct water from the middle and upper Aniene valley, even at the end of the flow path (Rome). This was due to the insulation of the water from the atmosphere: 84% of the flow path was below ground[10, 15.6] and above ground sections were composed of brick or concrete 0.3 - 1 m thick[our measurements, 12] around the entire cross-sectional perimeter of the aqueduct (including the roof). The access shafts (spaced more than 100 m apart at Roma Vecchia, Ashby 1935) seem to have been covered under normal operation[10]. Thus, the temperature variation of the water in the aqueduct would largely reflect that of the source water (the Aniene River). The late 20th century seasonal variation of the Aniene River (6 – 13.5^{o}C as outlined above) is comparable to the early Roman empire, when Frontinus stated that water was "still very cold and fresh" (*frigidissimus simul ac splendidissimus*, 93.3) at the aqueduct's intake. This 6 – 13.5^{o}C range results in a variation in kinematic viscosity of only +13/- 10% of the mean value. Interannual mean variation in source water temperature was likely to have been of a smaller magnitude, judging from the data obtained[39]. In Units 1 and 2 at Roma Vecchia, the wavelength has a total range over time of +/- 70-150% of the mean (see Supplementary Data Files) depending on location, once the ripples are fully established early in Unit 1.

S4.5 Wavelength Variation in Anio Novus Travertine Predominantly Forced by flow velocity

Changes in shear velocity in the Anio Novus aqueduct channel would have been primarily due to changes in flow velocity rather than other factors. For a given location, the channel slope, S, should change only slightly, if at all, over time, due to the effects of travertine deposition. With S constant, then by Equation 1, decreasing u^* will correspond to decreasing R_h. If u^* and R_h both decrease while mean wavelength increases, then by Equation 5 $\bar{u}$ must decrease, if B_L remains relatively constant. Since Re_c^* will be constant, B_L can only change by a maximum of +/- 0.75[Fig. 6 in 17], which is +/- 8 % of its value. This only produces a +/- 6% difference in the $\bar{u}$ calculated via Equation 6 for the "latest flow" at Roma Vecchia. In contrast, if the minimum and maximum wavelengths from Units 1 and 2 at Roma Vecchia (0.003 and 0.041 m, respectively, Fig. 6 and Supplementary Data File 3) are used in Equation 6 (along with the R_h for the "latest flow" at Roma Vecchia, Supplementary Table S2), then $\bar{u}$ varies by +/- 23%.

Regarding flowrate, if R_h decreases, then, by Equation 2, in a taller-than-wide rectangular channel, the cross-sectional flow area A must also decrease. When both $\bar{u}$ and A decrease, then by Equation 7, the flow rate Q must also decrease.

Thus, observed wavelength variation at Roma Vecchia likely has a significantly greater effect on $\bar{u}$ calculated by Equation 6 than variations in B_L (or temperature variation, as discussed above).

S4.6 Likely Time and Impact of Flow Rate Changes of Units 1 and 2 at Roma Vecchia.

In all three locations at Roma Vecchia, the travertine is deposited on an apparently pristine mortar floor surface (the t_0 surface; Figs. 4, 5) and there is no evidence of rebuilding of the channel or its mortar lining after the aqueduct's initial construction[12]. Given the hardness and strongly cemented nature of the travertine, it is highly unlikely that floor travertine could have been removed during maintenance at all three Roma Vecchia without causing visible mortar

damage [40]. This implies that Units 1 and 2 were deposited directly on the mortar soon after the 52 CE construction and opening of the Anio Novus.

At the best-preserved location (RF 140 m), Units 1 and 2 represent the lowest 8 cm of a 27-cm-thick floor deposit[16]. Assuming the Anio Novus operated for the longest possible time period (748 years: 52 - 800 CE: see section 4: Regional Setting), this 27 cm-thick accumulation implies a minimum mean depositional rate of 0.36 mm/year. If this mean rate holds for the first 8 cm, then that 8 cm was deposited over a maximum period of 222 years. However, the actual period is likely to significantly shorter, since the minimum rate assumes neither maintenance removal of travertine, which clearly occurred as they are visible at unconformities[40], nor periods where the aqueduct was out of use. Therefore, the mean depositional rate was likely significantly higher, especially when compared to the extremely high depositional rates observed in other natural travertine deposits[9,41].

The effects of the reduced flow rate inferred from the qualitative relationships are clear. Since the Anio Novus was the highest aqueduct in the Aniene valley, there would have been greater demand for its water upstream of Roma Vecchia, i.e. outside the city. More users stood to be affected by these reductions in its flowrate than by those of the other aqueducts. The well-known increased demand for perishable and luxury goods fuelled by Rome's increasing wealth and population during the first centuries BCE and CE would have increased pressure on the Anio Novus. Much land close to the city was given over to the production of high value-density products such as flowers, luxury meats, dairy and vegetables, which could not be imported from further afield[42]. Many of these products required irrigation to realise the greatest profit and irrigation technology was installed in Rome's hinterland for this purpose. Since private water concessions were given out by the emperor at this time[10], the wealthy and elite villa owners involved likely used their influence to gain water concessions for their fields (and elaborate gardens). These concessions would have been drawn above all from the Anio Novus, whose lower-quality water Frontinus considered more suitable for irrigation that that of the

spring-fed Aqua Claudia and Aqua Marcia. The concessions would have come, however, at the expense of Rome's large urban population. Tiber River lead contamination shows that the city's water distribution system, although probably contracting, was still near its peak at the time of the two flow reductions noted above[43].

S4.7 Climate-induced Variation in Aniene River Discharge

The Aniene River could probably have always supplied more water to the Anio Novus aqueduct than it could carry (2 m^3/s). For instance, in 1991, baseline flows in the Aniene River near the Anio Novus' entrance were always at least 5 - 8 m^3/s, with storms elevating the flow rate above 40 m^3/s at times[44]. The modern Aniene River has hydroelectric dams along its course, which is similar to the situation in antiquity when there were three dams at or above the intake to the Anio Novus, one of which fed the Anio Novus aqueduct[10,45]. The rainfall proxy evidence does not suggest that the Tiber basin was extremely dry during the 1st and 2nd century CE [46]. Hence it appears that flow in that period was comparable to the modern day and would rarely, if ever, be low enough to affect flows in the Anio Novus. This is especially likely when considering that Roman aqueduct intakes from rivers often incorporated a weir that diverted flow into the aqueduct[47], especially in times of low flow.

S5 Methods

S5.1 Aqueduct travertine Sampling Ssites

Galleria Egidio is located on the Tivoli loop, where little of the channel has been found, surveyed or accessed. The available evidence is largely consistent with a uniform flow assumption, however. The elevation of Galleria Egidio was not recorded during previous topographic surveys[48], but the mean gradient over the reach containing Galleria Egidio is

between 1 m/km and 1.5 m/km. This is one of the lowest gradients found along the entirety of the Anio Novus aqueduct. Given that a continual downhill gradient is required to prevent stagnation, there is little leeway for localized gradient variation along this reach or at Galleria Egidio[13]. The nearest known control points and junctions are about 1500 m upstream and 2600 m downstream. Surviving travertine deposits reach a maximum thickness of 32 cm on the channel floor and 39 cm on the walls[13].

The 140 m-long section of the Anio Novus at Roma Vecchia makes a strong case for uniform flow. It exhibits consistent geometric and building material construction, is straight, maintains a constant cross-sectional area, and has a consistent ~0.2% slope[12,16,48]. The geometry, cross-section and slope remain apparently constant for at least 1500 m upstream of this 140m-long section, as evidenced by below-[49] and above-[our survey and 50] ground remains, except for a 10° right turn (in the flow direction) 200 m upstream in the Via delle Capanelle (Supplementary Fig. S4). The same is true for 800 m downstream of this 140m-long section, except for a 20° left turn 20 meters downstream of the downstream 140 m sample site (Fig. 1B). In addition, the only known nearby control points or other features are a settling tank 600 m upstream and side branch which connected to a large nearby villa complex now known as "Sette Bassi". Since this branch was constructed in the mid-2nd century CE[12], it may not have been built until after the period when Units 1 and 2 were laid down, but was certainly built before "latest flow". Since, according to Ashby[12], this branch was supplied by the Anio Novus, it must have departed from the main line somewhere in the upstream portion of the 140-meter transect.

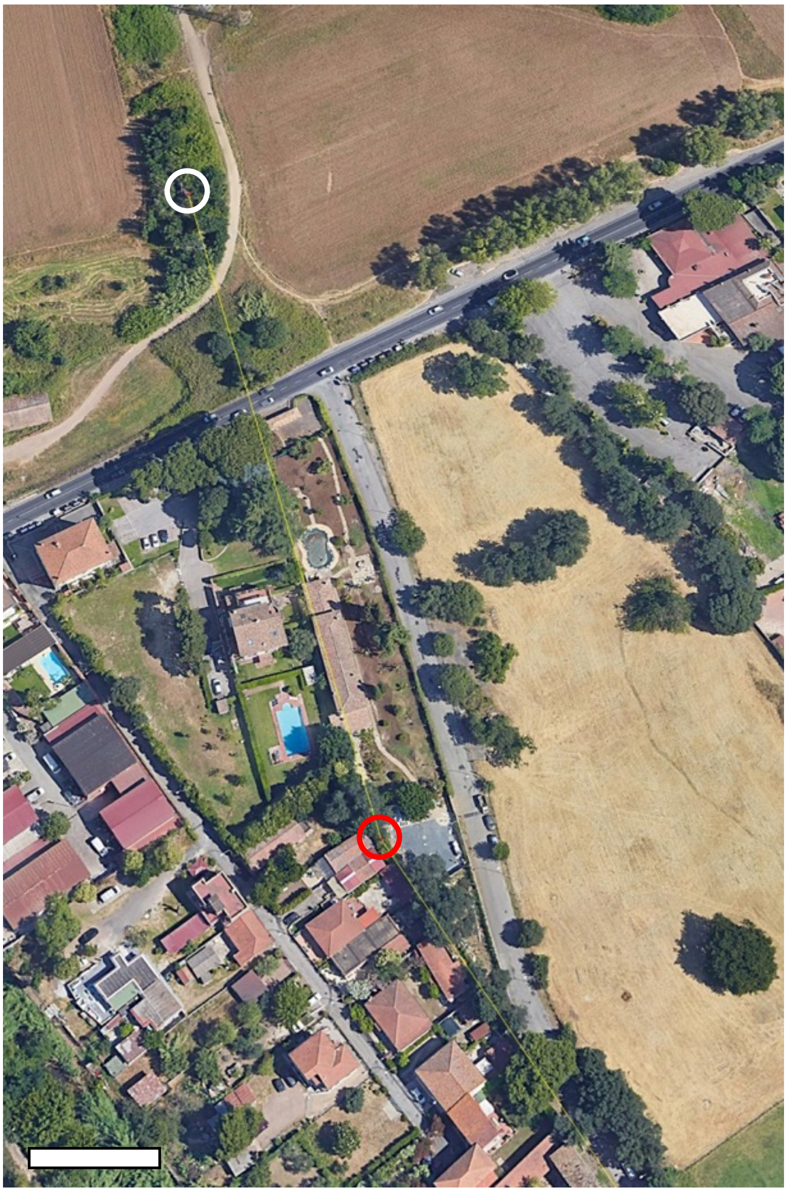

Supplementary Figure S4 The route of the Anio Novus (marked in yellow) upstream of RF 0 m (white circle) showing the 10º turn (red circle) 200 m upstream. Flow direction is from bottom to top (south to north). Base map: Google Earth (Imagery © 2021 Maxar Technologies, Map data © 2021). Scale bar: 30 m

Use of limestone deposits — called travertine — as a record of wetted perimeter, enabled the first estimation of actual flows in Rome's Anio Novus aqueduct at both Galleria Egidio and Roma Vecchia[13,16].

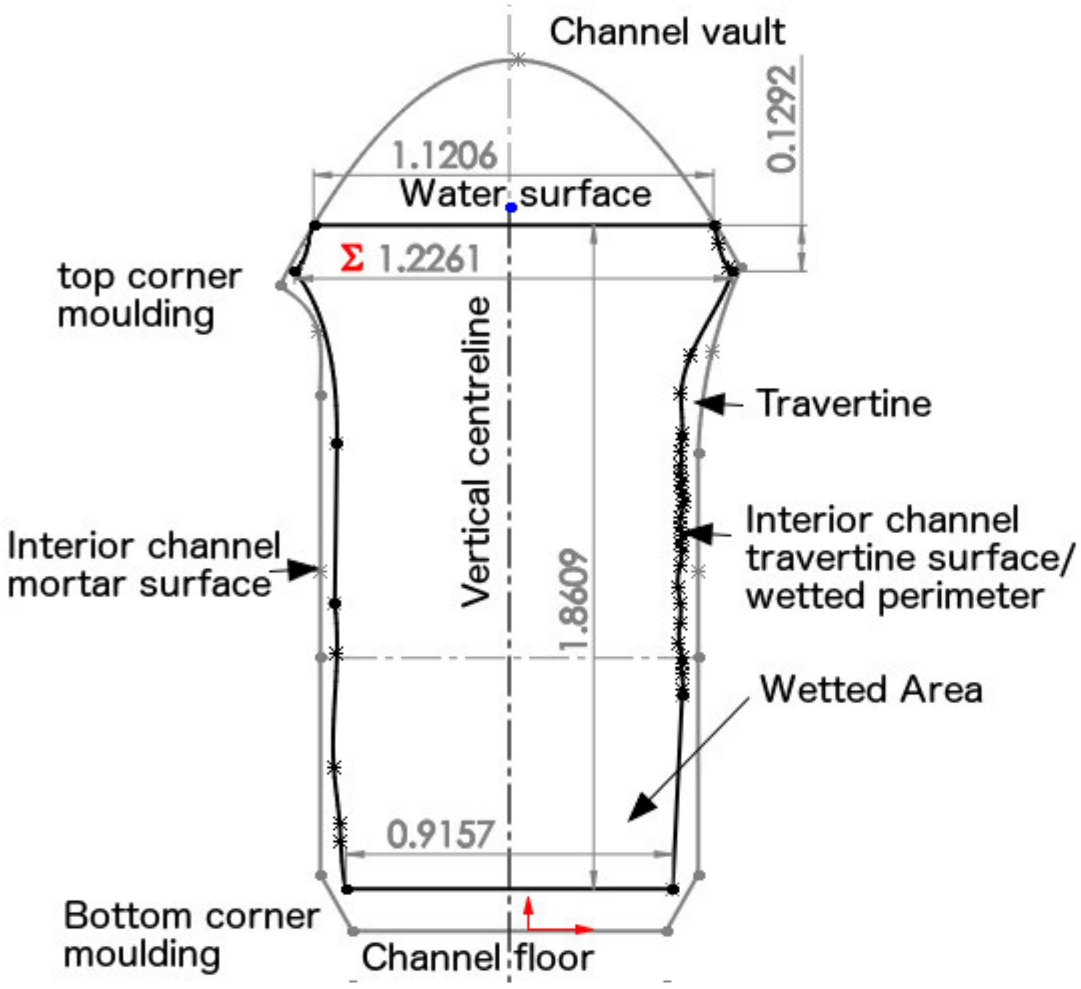

Supplementary Figure S5 Empiglione Bridge channel cross-section as reconstructed in SolidWorks 2016 (www.solidworks.com/) by Duncan Keenan-Jones, viewed from downstream (the same direction as the view in Fig. 2A, C). Asterisks show points measured using the Leica total station. All dimensions are in meters. Since the wall was damaged in the top left corner of the channel above the top corner moulding, the right-hand travertine surface was mirrored.

To minimize the effects of minor damage to the channel, resulting in possible unevenness of the cross section being displayed, average eastings and northings of the interior surface of

each wall (both shown in Supplementary Fig. S5) were taken and the distance between them calculated as a representation of the standard channel width.

S5.3 Aqueduct Travertine Ripple Characterization

MIPAV's livewire function produced specific coordinates for the horizons at irregular x intervals (where x is the coordinate in the downstream direction), permitting the Lomb-Scargle algorithm to be used on unevenly sampled datums. The Lomb-Scargle algorithm with WOSA was implemented using the REDFIT program[51] via the RED2CON interface (www.geo.uni-bremen.de/geomod/staff/mschulz) between MATLAB and REDFIT. The highest wavenumber analysed was always the Nyquist wavenumber (which is equal to half the average sampling rate and is implemented by setting the "hifac" parameter to 1). The oversampling factor was set to 4.0. There was no prescribed value for ρ (the average autocorrelation coefficient), except where problems with the significance testing in REDFIT required it, as suggested by the creators of REDFIT[52]. The Fourier transforms sometimes returned significant wavelengths longer than those recognizable during ground truthing.

Four amplitude proxies were tested against ground-truthed amplitude (which was the mean of 2 measurements at each horizon). These were calculated in the following way. When exported from MIPAV, the digitised ripple horizons were located entirely above the x-axis (i.e. all y-values were positive). The difference between the largest and smallest y-values (i.e. the vertical range) was divided by two to give the Vertical Range (no baseline correction) proxy (shown as blue triangles in Supplementary Fig. S6). The horizons were then baseline corrected[53]. First the horizon was centred around the x-axis by subtracting the value of a 4th-order polynomial (fitted to the horizon) where its second differential was equal to zero. A fitted sinusoidal baseline was then subtracted, to remove a ripple scale larger than the sample, if one existed. The amplitude of this sinusoidal baseline was another (shown as red triangles in Supplementary Fig. S6). The vertical range was then calculated again to give the Vertical Range (baseline corrected) proxy (shown as black triangles in Supplementary Fig. S6). Then

mean of the absolute value of the horizon was calculated to determine the mean displacement proxy (shown as grey triangles in Supplementary Fig. S6).

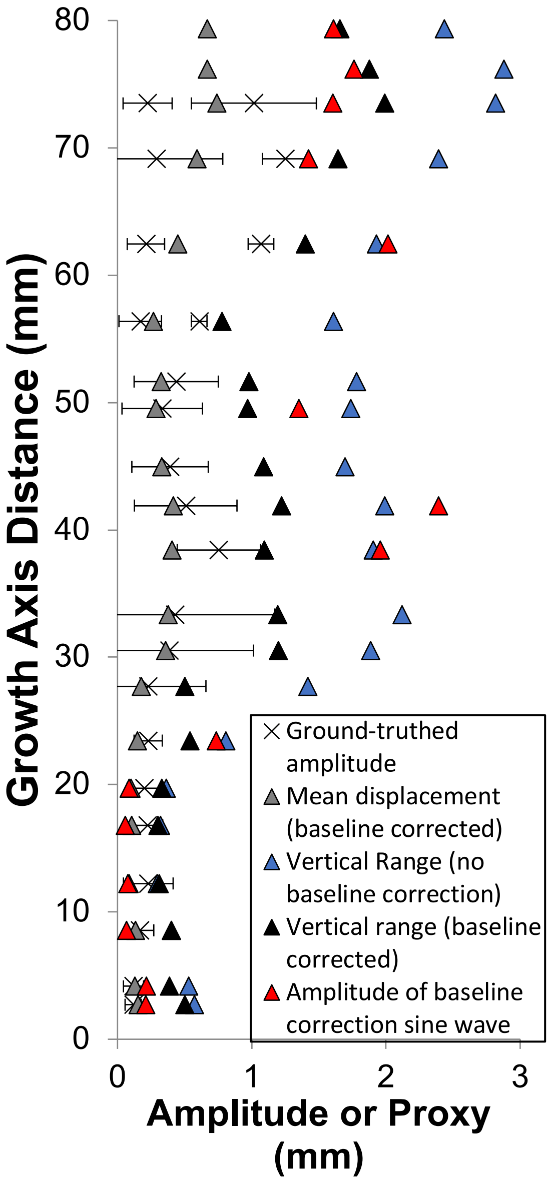

Supplementary Figure S6 Comparison of amplitude proxies with ground truthing for RF 140 m (Supplementary Data File 3). Several points of the amplitude of baseline correction sine wave plot far off the graph. The distance in the direction of growth from the zero-time surface (t_0) is plotted on the y-axis.

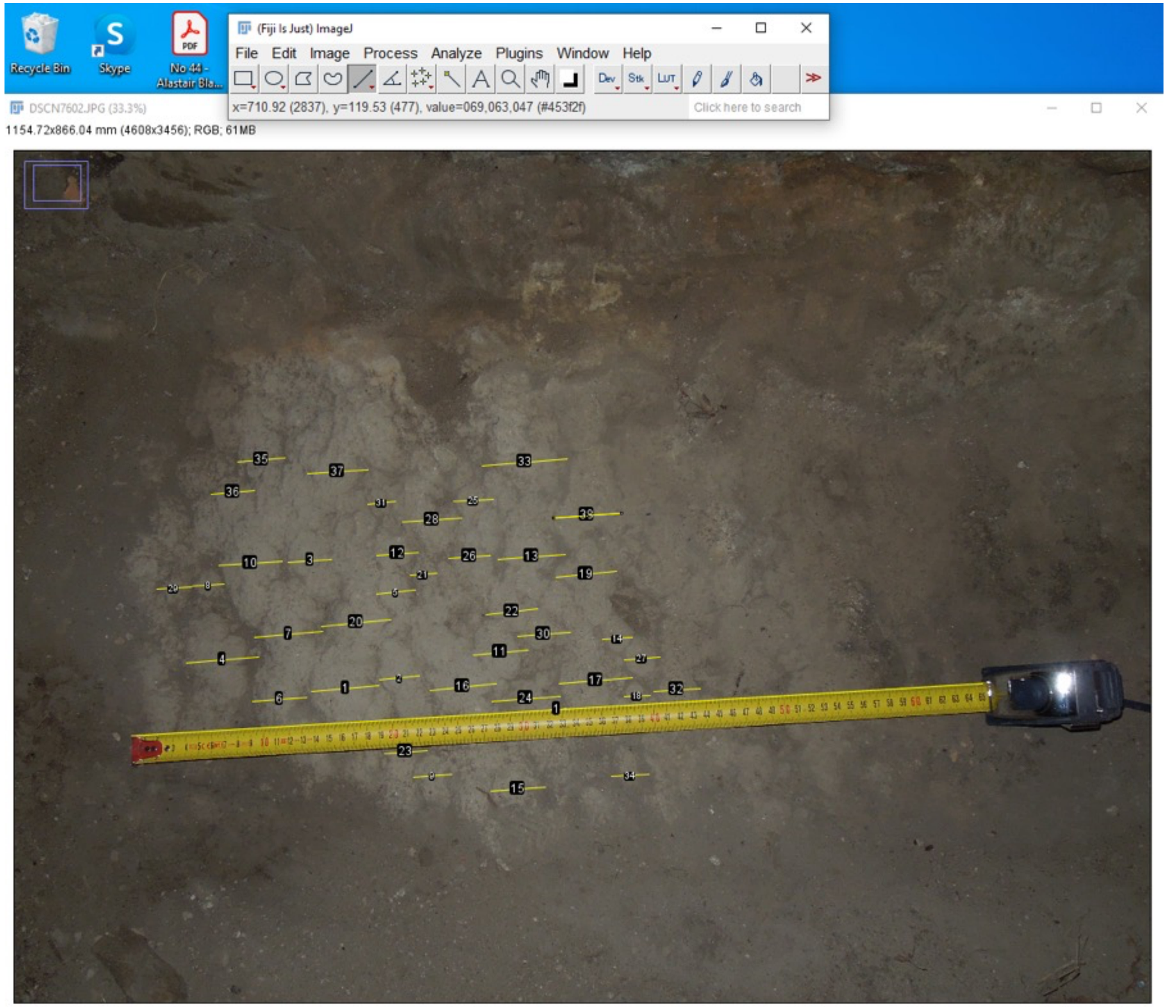

Supplementary Figure S7 Plan view of ripples on the floor at Galleria Egidio "early flow", showing the wavelength measurement in ImageJ. These ripples are exposed a few meters downstream of the Galleria Egidio measuring section[13]. The tape measure shows cm.

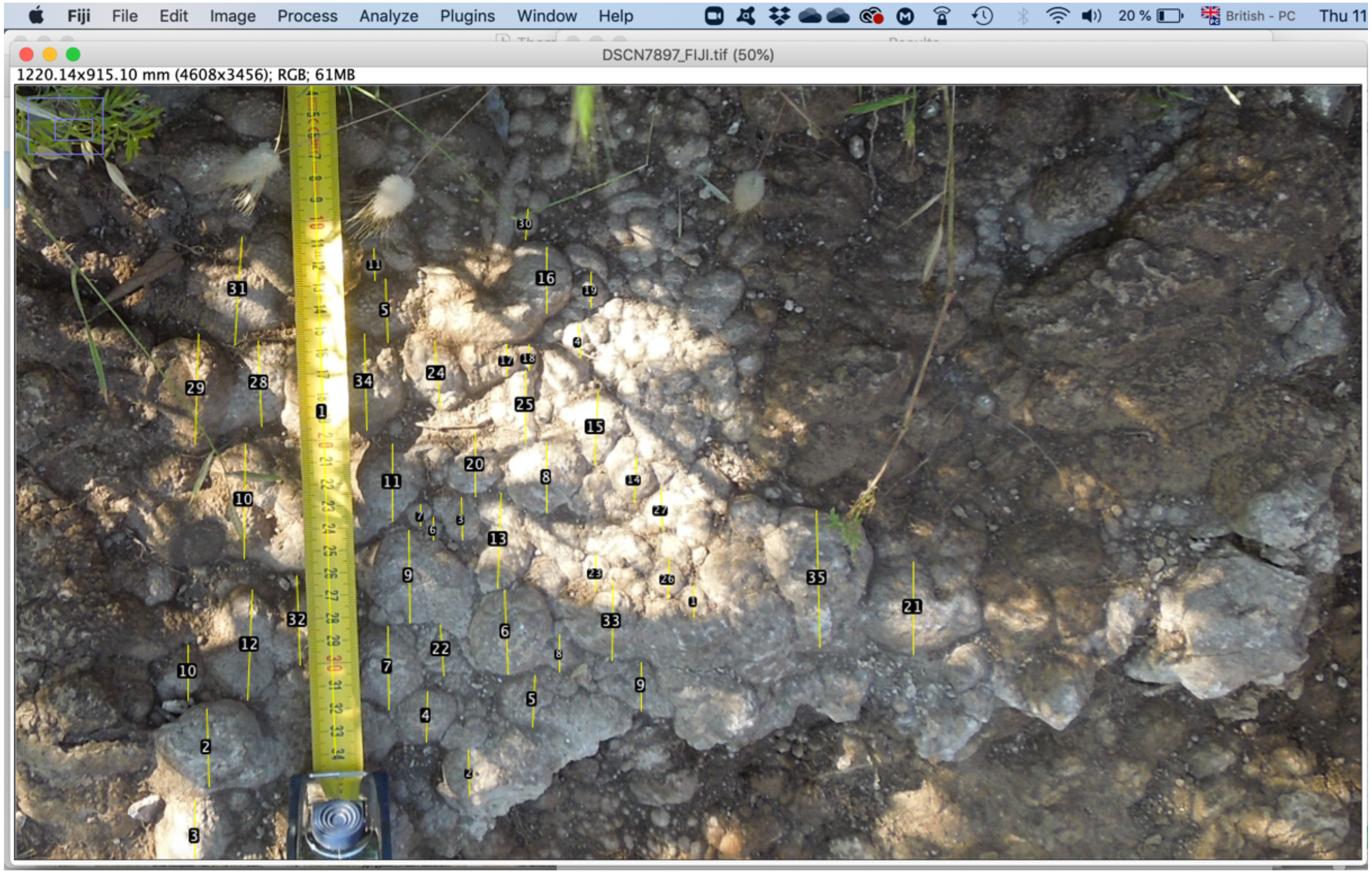

Supplementary Figure S8 Plan view of ripples on the floor at Roma Vecchia RF 140 m, showing the wavelength measurement of the "latest flow" surface only in ImageJ. Unmeasured ripples belong to lower depositional surfaces older than the "latest flow". The tape measure shows cm.

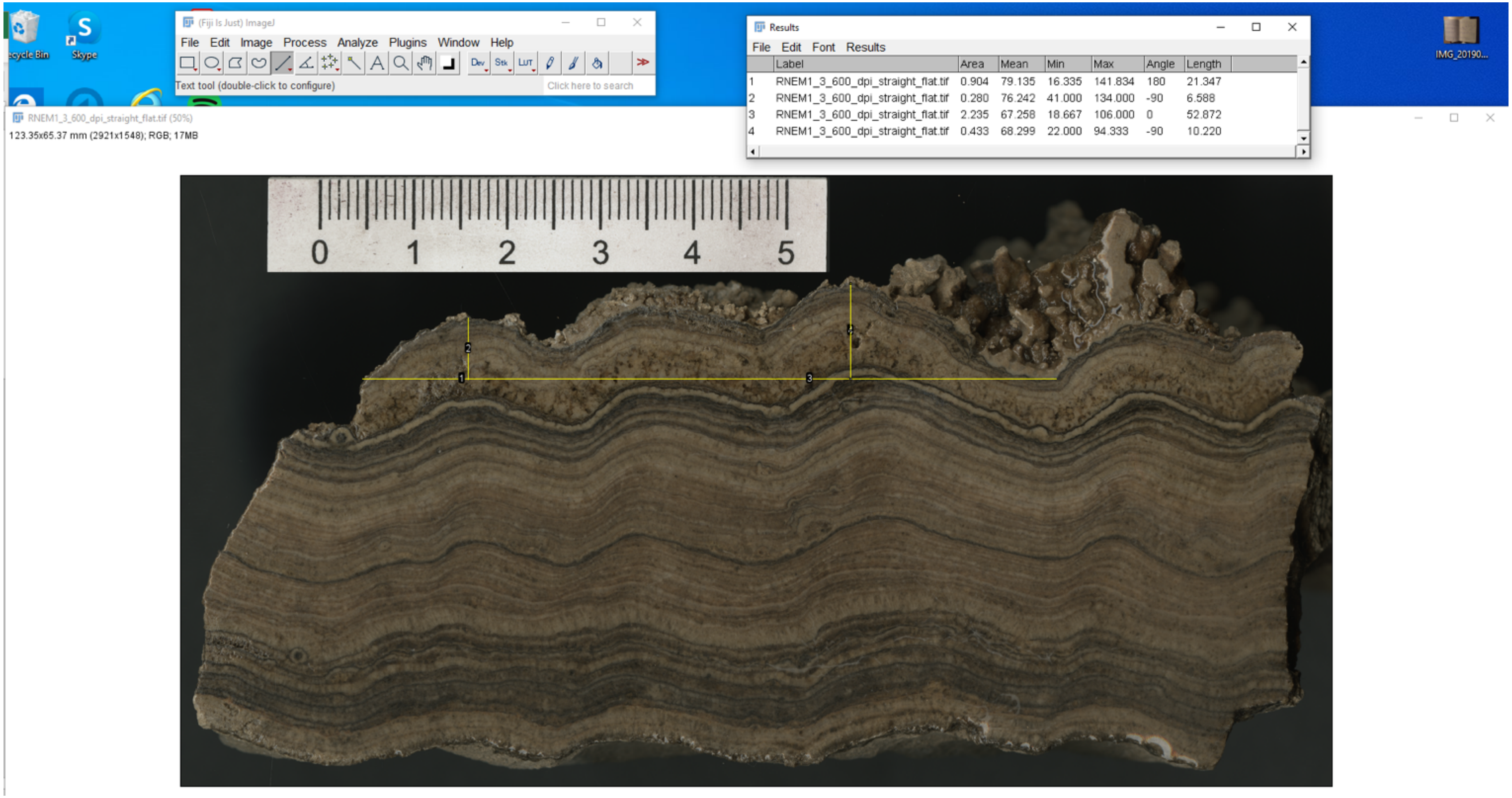

Supplementary Figure S9 Black and white polished cross-section view of ripples in hand sample at Empiglione Bridge "latest flow", showing the wavelength and amplitude measurement of ripples in ImageJ. Length units are in millimeters as are scale divisions. Flow direction is from

left to right. Sample was matched to the latest flow at Empiglione Bridge by matching the characteristic "popcorn" surface morphologies visible in the channel (Fig. 2C) and on the top of the sample cross-section. These "popcorn" growths are very different from the rest of the sample and likely formed during unusual flow conditions related to the breakdown of the aqueduct.

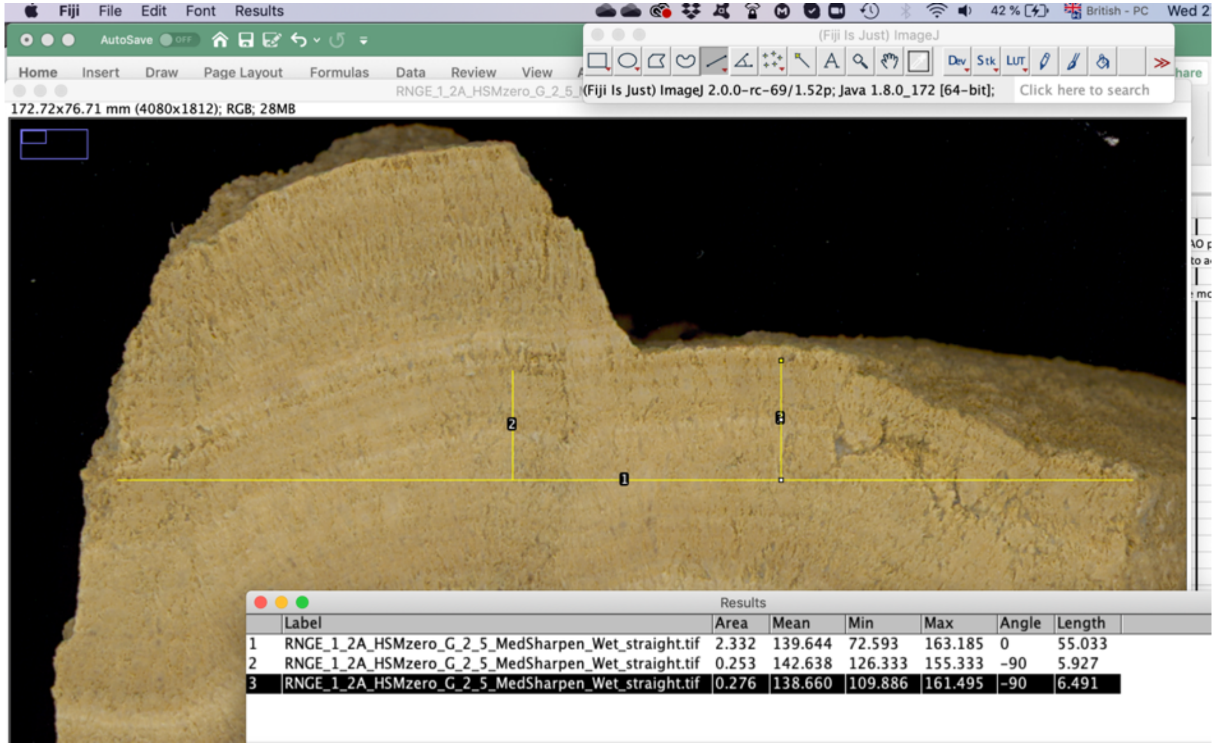

Supplementary Figure S10 Polished cross-section view of ripples on the wall at Galleria Egidio "early flow", showing the wavelength and amplitude measurement in ImageJ. Measurement 3 was used for amplitude since it was bigger than measurement 2. Length units are in millimeters. Flow directions is from left to right.

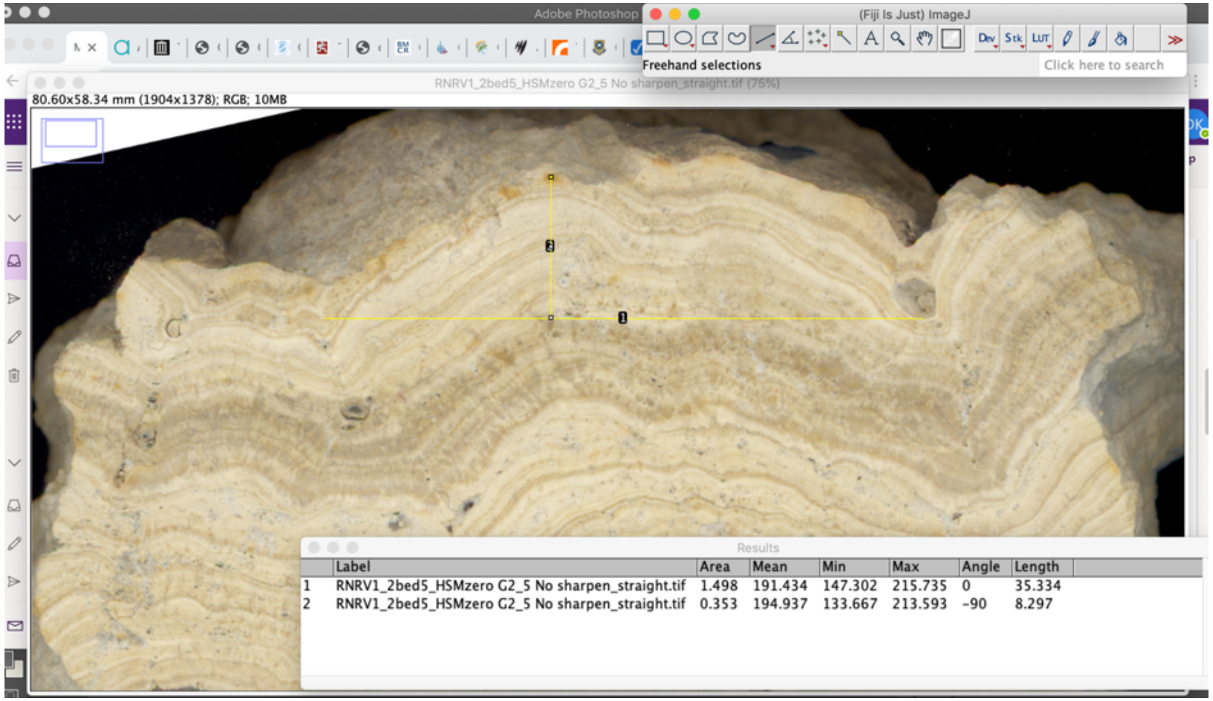

Supplementary Figure S11 Polished cross-section view of ripples on floor at Roma Vecchia "latest flow", showing the wavelength and amplitude measurement in ImageJ. Length units are in millimeters. Flow directions is from left to right.

S5.4 Calculation of Critical Shear Reynolds Number from Aqueduct Travertine Ripples

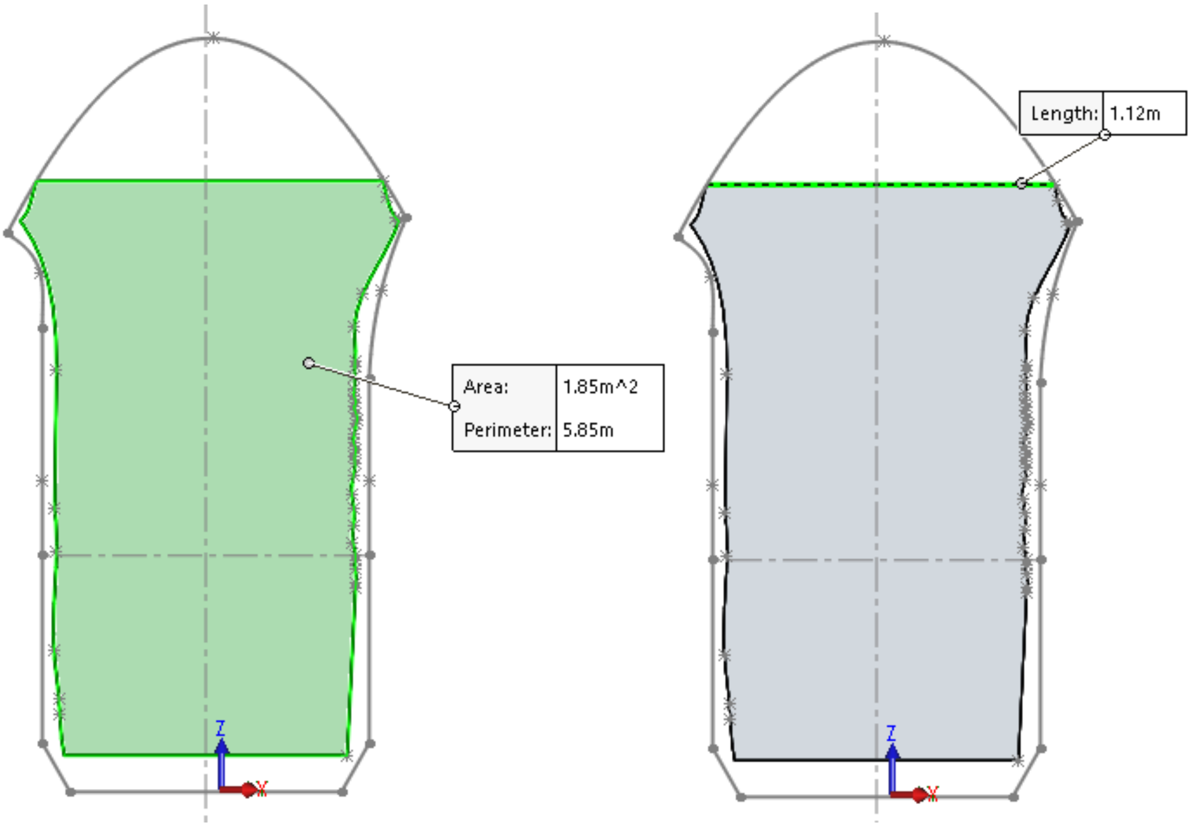

Supplementary Figure S12 Calculation of wetted area and perimeter of the Empiglione Bridge "latest flow" channel (viewed from downstream) modelled in SolidWorks 2016 (www.solidworks.com/) by Duncan Keenan-Jones. The value for the perimeter calculated by SolidWorks included the water surface (Left), so the water surface length was measured in SolidWorks (Right) and subtracted.

Data from Agenzia Regionale per la Protezione Ambientale del Lazio (ARPA Lazio, http://www.arpalazio.gov.it/, Supplementary Fig. S11B) and Bono and Percopo's[44] monitoring of the modern Aniene River showed that modern Aniene water temperature varies between 6 and 13.5 °C seasonally. Hence, a temperature of 10 °C was used to calculate the mass density ρ (997.7 kg/m^3) and dynamic viscosity μ (0.0013 Ns/m^2) of water, using the formulations by Pátek et al.[54]. The water kinematic viscosity (ν = 1.31 +/- 0.12 x 10^{-6} m^2/s) was computed as μ/ρ.

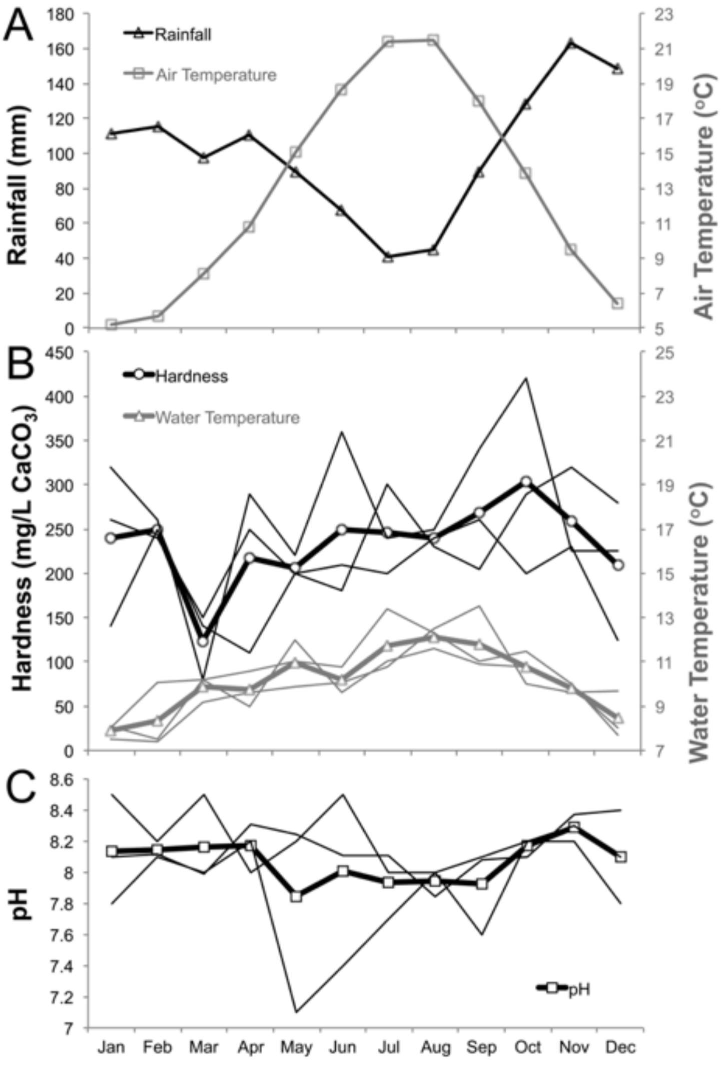

Supplementary Figure S13 Modern climate and hydrogeochemistry of the Aniene River basin. A. Historic rainfall and temperature averages at Subiaco. 85 years of rainfall data and 56 years of temperature data collated by the Ufficio Idrografico e Mareografico, Regione Lazio[55]. B & C. Aniene River water chemistry, 2006-2008.

(Agenzia Regionale per la Protezione Ambientale del Lazio, http://www.arpalazio.net/). A thicker mean line overlies the data from the individual years.

Supplementary Video Captions

Supplementary Video S1. Sequence of Micro-CT slices from a sample from the RF 0 m location at Roma Vecchia, proceeeding from the time-zero (t_0) oldest surface to youngest surface, and then back to the t_0 surface. The arrow at the beginning indicates the flow direction. A cross section (equivalent to Supplementary Figure 1A) at the side of the slices shows the location of each slice within the stratigraphy.

Supplementary Video S2. Sequence of Micro-CT slices from a sample from the RF 0 m location at Roma Vecchia, proceeeding from the time-zero (t_0) oldest surface to youngest surface, and then back to the t_0 surface. The arrow at the beginning indicates the flow direction.

References

1. Camporeale, C. & Ridolfi, L. Ice ripple formation at large Reynolds numbers. *J. Fluid Mech.* **694**, 225–251 (2012).
2. Yokokawa, M. *et al.* Cyclic steps on ice. *J. Geophys. Res. Earth Surf.* **121**, 1023–1048 (2016).
3. Ashton, G. D. & Kennedy, J. F. Ripples on Underside of River Ice Covers. *J. Hydraul. Div.* **98**, 1603–1624 (1972).
4. Camporeale, C. & Ridolfi, L. Hydrodynamic-Driven Stability Analysis of Morphological Patterns on Stalactites and Implications for Cave Paleoflow Reconstructions. *Phys. Rev. Lett.* **108**, 238501 (2012).
5. Curl, R. L. Scallops and Flutes. *Trans. Cave Res. Group G. B.* **7**, 121–160 (1966).

6. Goodchild, M. F. & Ford, D. C. Analysis of Scallop Patterns by Simulation under Controlled Conditions. *J. Geol.* **79**, 52–62 (1971).
7. Andritsos, N. & Karabelas, A. J. Calcium carbonate scaling in a plate heat exchanger in the presence of particles. *Int. J. Heat Mass Transf.* **46**, 4613–4627 (2003).
8. Florsheim, J. L. *et al.* Basin-scale and travertine dam-scale controls on fluvial travertine, Jiuzhaigou, southwestern China. *Geomorphology* **180–181**, 267–280 (2013).
9. Fouke, B. W. Hot-spring Systems Geobiology: abiotic and biotic influences on travertine formation at Mammoth Hot Springs, Yellowstone National Park, USA. *Sedimentology* **58**, 170–219 (2011).
10. Frontinus, S. I. *De aquaeductu urbis Romae*. (Cambridge University Press, 2004).
11. Taylor, R. M. *Public needs and private pleasures : water distribution, the Tiber river and the urban development of ancient Rome*. ('L'Erma' di Bretschneider, 2000).
12. Ashby, T. *The aqueducts of ancient Rome*. (Oxford University Press, 1935).
13. Motta, D., Keenan-Jones, D., Garcia, M. H. & Fouke, B. W. Hydraulic Evaluation of the Design and Operation of Ancient Rome's Anio Novus Aqueduct. *Archaeometry* **59**, 1150–1174 (2017).
14. Fernandez, R., Best, J. & López, F. Mean flow, turbulence structure, and bed form superimposition across the ripple-dune transition. *Water Resour. Res.* **42**, n/a-n/a (2006).
15. Springer, G. & Hall, A. Uncertainties associated with the use of erosional cave scallop lengths to calculate stream discharges. *Int. J. Speleol.* **49**, 27–34 (2020).
16. Keenan-Jones, D. C., Motta, D., Garcia, M. H. & Fouke, B. W. Travertine-based estimates of the amount of water supplied by ancient Rome's Anio Novus aqueduct. *J. Archaeol. Sci. Rep.* **3**, 1–10 (2015).
17. Blumberg, P. N. & Curl, R. L. Experimental and theoretical studies of dissolution roughness. *J. Fluid Mech.* **65**, 735–751 (1974).

18. Allen, J. R. L. Transverse erosional marks of mud and rock: their physical basis and geological significance. *Sediment. Geol.* **5**, 167–385 (1971).

19. Villien, B., Zheng, Y. & Lister, D. Surface Dissolution and the Development of Scallops. *Chem. Eng. Commun.* **192**, 125–136 (2005).

20. Hammer, Ø. *et al.* Calcite precipitation instability under laminar, open-channel flow. *Geochim. Cosmochim. Acta* **72**, 5009–5021 (2008).

21. Hanratty, T. J. Stability of Surfaces that are Dissolving or Being Formed by Convective Diffusion. *Annu. Rev. Fluid Mech.* **13**, 231–252 (1981).

22. Baas, J. H., Best, J. L. & Peakall, J. Predicting bedforms and primary current stratification in cohesive mixtures of mud and sand. *J. Geol. Soc.* **173**, 12–45 (2016).

23. Perillo, M. M., Prokocki, E. W., Best, J. L. & García, M. H. Bedform Genesis from Bed Defects under Unidirectional, Oscillatory and Combined Flows. *J. Geophys. Res. Earth Surf.* 2014JF003167 (2014) doi:10.1002/2014JF003167.

24. Perillo, M. M. *et al.* A unified model for bedform development and equilibrium under unidirectional, oscillatory and combined-flows. *Sedimentology* **61**, 2063–2085 (2014).

25. Perillo, M. M., Best, J. L. & Garcia, M. H. A New Phase Diagram for Combined-Flow Bedforms. *J. Sediment. Res.* **84**, 301–313 (2014).

26. Bird, A. J., Springer, G. S., Bosch, R. F. & Curl, R. L. Effects of surface morphologies on flow behavior in karst condjuits. in *Proceedings of the 15th Int. Congress of Speleology, Kerrville, Texas, USA, July 19-26, 2009* vol. 3 1417–1421 (2009).

27. Gudmundsson, J. S. & Bott, T. R. Deposition of silica from geothermal waters on heat transfer surfaces. *Desalination* **28**, 125–145 (1979).

28. Thorsness, C. B. & Hanratty, T. J. Stability of dissolving or depositing surfaces. *AIChE J.* **25**, 697–701 (1979).

29. Curl, R. L. Deducing Flow Velocity in Cave Conduits from Scallops. *Natl. Speleol. Soc. Bull.* **36**, 1–5 (1974).

30. Camporeale, C. & Ridolfi, L. Ice ripple formation at large Reynolds numbers. *J. Fluid Mech.* **694**, 225–251 (2012).

31. Ueno, K. Ripples on icicles and stalactites. *RIMS Kokyuroku Bessatsu* **B3**, 101–119 (2007).

32. Ueno, K., Farzaneh, M., Yamaguchi, S. & Tsuji, H. Numerical and experimental verification of a theoretical model of ripple formation in ice growth under supercooled water film flow. *Fluid Dyn. Res.* **42**, 025508 (2010).

33. Ogawa, N. & Furukawa, Y. Surface instability of icicles. *Phys. Rev. E* **66**, 041202 (2002).

34. Meakin, P. & Jamtveit, B. Geological pattern formation by growth and dissolution in aqueous systems. *Proc. R. Soc. Math. Phys. Eng. Sci.* **466**, 659–694 (2010).

35. van Rijn, L. C. Equivalent Roughness of Alluvial Bed. *J. Hydraul. Div.* **108**, 1215–1218 (1982).

36. Garcia, M. H. *Sedimentation engineering: processes, measurements, modeling, and practice*. (American Society of Civil Engineers, 2008).

37. Chow, V. T. *Open-channel hydraulics*. (McGraw-Hill, 1959).

38. Ackers, P. Experiments on Small Streams in Alluvium. *J. Hydraul. Div.* **90**, 1–37 (1964).

39. Büntgen, U. *et al.* 2500 Years of European Climate Variability and Human Susceptibility. *Science* **331**, 578–582 (2011).

40. Sivaguru, M. *et al.* Impact of Diagenetic Alteration on Travertine Crystalline Shrubs, Biofilm Laminae and Ripple Marks in the Anio Novus Aqueduct of Ancient Rome. in *From the Guajira Desert to the Apennines, and from the Sardinia/Corsica Microplate to the Killer Asteroid: Honoring the Career of Walter Alvarez* (eds. Bartley, J. K., Boyce, C. K. & Kah, L. C.) (Geological Society of America, 2021).

41. Pentecost, A. *Travertine*. (Springer, 2005).

42. Ronin, M. Funding Irrigation. Between Individual and Collective Investments. in *Capital, Investment, and Innovation in the Roman World Section: Capital, Investment, and*

Innovation in the Roman World (eds. Erdkamp, P., Verboven, K. & Zuiderhoek, A.) 225–245 (Oxford University Press, 2020).

43. Delile, H. *et al.* Rome's urban story inferred from lead-contaminated waters trapped in its ancient harbor basins. *Proc. Natl. Acad. Sci. U. S. A.* **114**, 10059–10064 (2017).
44. Bono, P. & Percopo, C. Flow dynamics and erosion rate of a representative karst basin (Upper Aniene River, Central Italy). *Environ. Geol.* **27**, 210–218 (1996).
45. Smith, N. A. F. The Roman Dams of Subiaco. *Technol. Cult.* **11**, 58–68 (1970).
46. Keenan-Jones, D. C. *et al.* Improved Tiber River Floods dataset as a new Central Italian rainfall proxy since Roman times. (In Preparation).
47. Hodge, A. T. *Roman Aqueducts and Water Supply*. (Duckworth, 2002).
48. Reina, V., Corbellini, G. & Ducci, G. *Livellazione degli antichi acquedotti romani*. (Tipografia della R. Accademia dei Lincei, 1917).
49. Blanco, D. & Sebastiani del Grande, P. Via Lucio Mariani (Gregna Sant'Andrea). Resti di acquedotti e tracciati viari d'epoca romana (Municipio X). *Bull. Della Comm. Archeol. Comunale Roma* **117**, 380–384 (2016).
50. Ceccherelli, A. Dalle piscine limarie al castello terminale. in *Gli acquedotti Claudio e Aniene Nuovo nell'area della Banca d'Italia in via Tuscolana* 33–47 (Istituto Poligrafico e Zecca dello Stato, 2001).
51. Schulz, M. & Mudelsee, M. REDFIT: estimating red-noise spectra directly from unevenly spaced paleoclimatic time series. *Comput. Geosci.* **28**, 421–426 (2002).
52. Schulz, M. *REDFIT 3.8e. Estimating red-noise spectra directly from unevenly spaced paleoclimatic time series. Manual (November 2010)*. http://www.geo.uni-bremen.de/geomod/staff/mschulz/software/redfit38e.zip (2010).
53. Chen, A. S.-H. & Morris, S. W. On the origin and evolution of icicle ripples. *New J. Phys.* **15**, 103012 (2013).

54. Pátek, J., Hrubý, J., Klomfar, J. & Součková, M. Reference Correlations for Thermophysical Properties of Liquid Water at 0.1MPa. *J. Phys. Chem. Ref. Data* **38**, 21–29 (2009).

55. Ufficio Idrografico e Mareografico, Regione Lazio. *Elaborazione Dei Dati Termo - Pluvio - Idrometrici Osservati Sul Territorio Della Regione Lazio Nel Periodo Gennaio 2007 - Febbraio 2008*. http://www.idrografico.roma.it/documenti/Elaborazione%20termo%20pluvio%20idro%20gen%2007%20-%20feb%2008.pdf (2008).
